# Topographical transition of submicron pillar array of azo molecular glass induced by circularly polarized light

**DOI:** 10.1038/s41598-021-86794-y

**Published:** 2021-04-01

**Authors:** Zenan Wang, Chungen Hsu, Xiaogong Wang

**Affiliations:** Department of Chemical Engineering, Laboratory of Advanced Materials (MOE), Tsinghua University, Beijing, 100084 People’s Republic of China

**Keywords:** Materials science, Optics and photonics

## Abstract

The well-aligned submicron patterns on surfaces have attracted wide attention from scientific curiosity to practical applications. Understanding their formation and transition is highly desirable for efficient manufacture of the patterns for many usages. Here, we report a unique observation on self-organized topographical transition of submicron pillar array of an azo molecular glass, induced by irradiation with circularly polarized light. During gradual erasure of the patterns upon exposure to the light, which is a property of this material, a new set of pillars unexpectedly emerge with new one in middle of each triangle cell of the original array. The highly regular pillar array with triple area density is formed and finally stabilized in the process, as revealed by thorough investigation reported here. This unusual observation and its rationalization will be of benefit for deep understanding of the light–matter interaction and can be expected to be applied in different areas.

## Introduction

The formation of well-aligned submicron patterns on surfaces extending over the macroscopic scale has aroused tremendous research enthusiasm for interests from fundamental understanding to various applications^1–4^. Unique surface patterns found on the feathers of birds, wings of butterflies and other living creatures have been a subject of intensive study for many years^5^. Artificially patterned surfaces have been widely used in microelectronics, optics, photonics, flexible optoelectronics, and bio-mimicking substances^6–8^. Due to the consistent technological innovations, micro/nanoscale patterns over large-area can now be well fabricated by different methods, such as photolithography^6^, laser interference lithography (LIL)^9^, the electron-beam lithography (EBL)^10^, focused ion beam (FIB) lithography^11^, nanoimprint lithography (NIL)^12,13^, and soft lithography^14,15^. Among them, the lithographic methods based on light irradiation show some unique advantages for large-scale applications^6,9^. In recent years, azo polymers and azo molecular glasses (polymers and molecular amorphous materials containing azo chromophores) have been intensively investigated for fabricating erasable surface-relief-gratings and other surface structures through LIL^16–26^. The surface pattern formation is caused by the mass transfer in the direction of the electric vibration of polarized light, as driven by the trans–cis isomerization of azo chromophores^27–30^. Distinct from conventional LIL using a photoresist, not only the intensity modulation of the light wave but also the local polarization variation can be employed to fabricate various complex patterns on surfaces, and the formed patterns can be erased by heating or irradiation with circularly polarized light^19–22^. Another intriguing property of this type of materials is related to the irradiation with a single laser beam, which can generate self-structured periodic patterns on the film surfaces of azo polymers and azo molecular glasses^31–34^. At the current stage, the forming machinery of the self-structured patterns still remains a mystery.

Azo molecular glass is a type of low-molecular-weight organic substance, which can exist in stable amorphous state below the glass transition temperature (*T*_g_)^35–37^. Being free from long-chain entanglements, they are usually more efficient to form surface patterns through photoinduced mass transfer compared with their polymeric counterparts^24,38,39^. Various surface patterns can be readily inscribed on thin films of an azo molecular glass under irradiation with interfering laser beams or a single laser beam^24,33,34,37–39^. In addition to the superior photoresponsive properties, some azo molecular glasses have recently been found to be suitable for the soft-lithographic hot embossing^40^. This property can be employed to fabricate well-defined structures such as hexagonal arrays of submicron pillars on a substrate. Distinct from common practice using thin films for surface patterning, the ordered submicron-pillar array made of an azo molecular glass has rarely been studied for the photoinduced pattern formation and transition. In contrast to the patterns predesigned and fabricated, self-organized topographical transition of submicron pillar array through light irradiation can pave a new avenue to explore fascinating property of this type of materials for surface patterning. However, to our knowledge, the self-organized transition of well-defined submicron patterns under the light irradiation has not been reported in the literature yet.

In this study, the hexagonal arrays of submicron pillars made of an azo molecular glass (IA-Chol) were prepared by the soft-lithographic hot embossing, and then exposed to circularly polarized light (λ = 532 nm) from a semiconductor laser. It was expected that the light irradiation could cause the mass transfer along the direction of the electric vibration of the polarized light, as observed by previous studies^28–30^, which would consequently erase the relief structures on the surface. To our surprise, instead of erasure of the surface structures, the pillar arrays showed unique self-organized topographical transition from the original pillar arrays to new hexagonal arrays of submicron pillars with a triple area density. Under the light irradiation, a new pillar gradually appeared in the middle of each triangle cell of the original pillars, where the height and diameter of the original pillars decreased accordingly. When the light irradiation continued, the final steady state was achieved to show the well-defined hexagonal array with triple area density, where the pillars were highly uniform but their sizes were different from the original ones. The self-organized topographical transition was thoroughly investigated by microscopic observations, and the results with rationalization of the mechanism are presented below in detail.

## Results

### Self-organized transition induced by light

**Figure 1:**
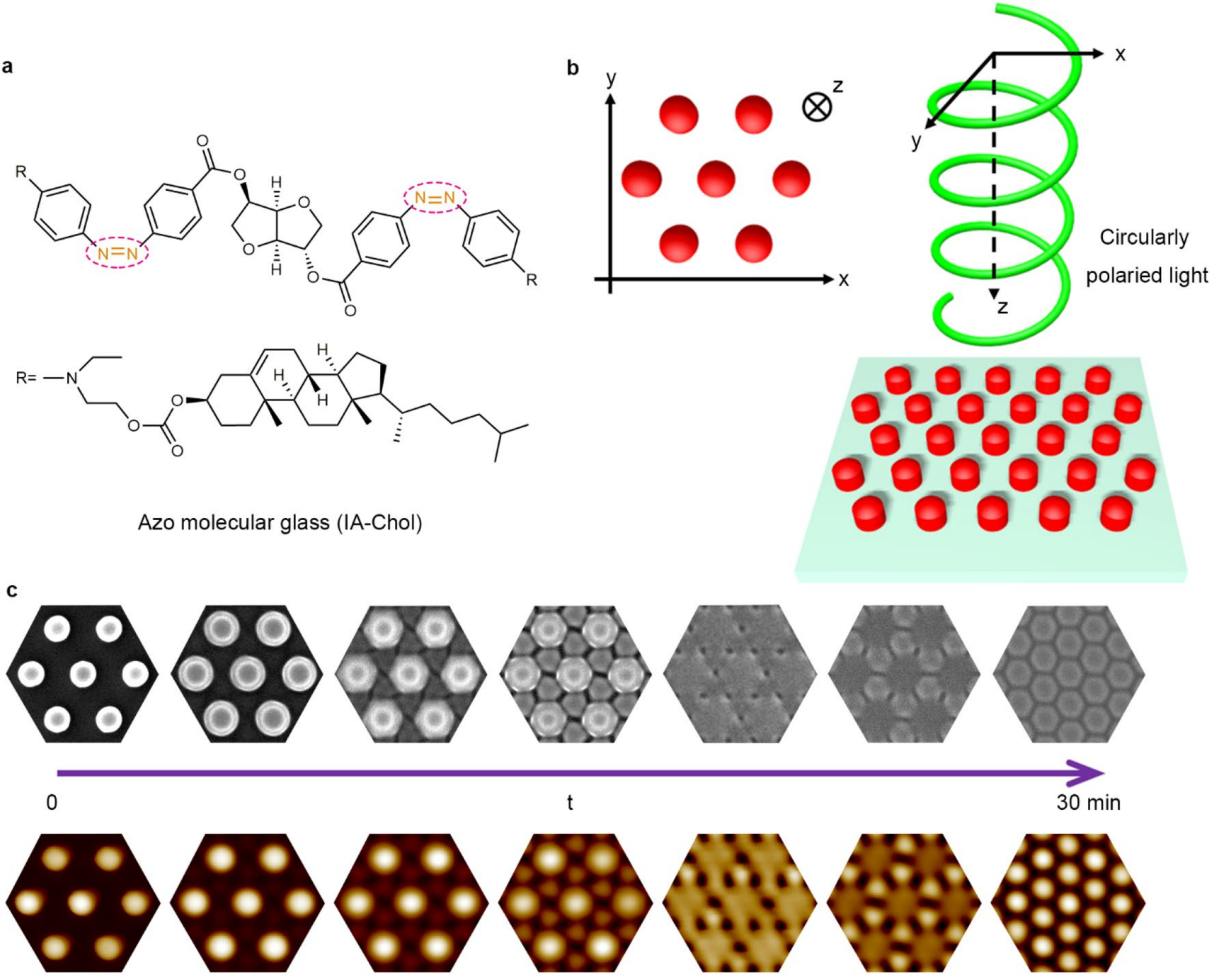
Molecular structure, irradiation condition and the surface topographical transition of submicron pillar arrays. (**a**) The chemical structure of the azo molecular glass (IA-Chol). (**b**) Schematic of the IA-Chol submicron pillar arrays irradiated by the right-hand circularly polarized light. (**c**) The SEM (top) and AFM (bottom) images of the surface topographical transition of IA-Chol pillar arrays induced by the right-hand circularly polarized light. From left to right, the exposure time was 0, 4 min, 6 min, 8 min, 10 min, 14 min and 30 min, respectively.

Figure 1a shows the chemical structure of the azo molecular glass (IA-Chol), which has an isosorbide moiety as the core bearing two *push*–*pull* type azo chromophores in middle and cholesteryl groups on the periphery. The synthesis and characterization of this compound are given in Experimental part and the Supplementary of this article. The *push*–*pull* type azo chromophore contained in IA-Chol is also called the pseudo-stilbene type azo chromophore. As the π–π*and n–π* transition bands for this type of molecules are overlapped in the visible light range, the light activating trans-to-cis isomerization will also activate the cis-to-trans isomerization, which then causes repeated trans–cis-trans isomerization cycle under visible light irradiation and consequently the efficient mass transfer of the materials containing this type of chromophores^18–21^. IA-Chol is an amorphous solid at the room temperature with the glass transition temperature (*T*_g_) of 69 °C and the decomposition temperature (*T*_d_) of 276 °C^40^.

The hexagonal periodic submicron-pillar arrays of IA-Chol were fabricated from its films by hot embossing with an elastomeric poly(dimethylsiloxane) (PDMS) mold. In the process, the thin solid films of IA-Chol with thickness in the range from 300 to 400 nm were obtained by spin-coating of the DMF solution on the clean glass slides and drying appropriately. The hot embossing was carried out by press the PDMS mold on the films under the temperature of 120 °C for 1 h. Supplementary Figure 1 shows the UV–vis spectral variations of IA-Chol under visible light irradiation and thermal relaxation in the dark for its DMF solution, solid thin film and submicron-pillar array. The much lower cis isomer content compared with a typical azobenzene-type compound in the steady state evidences the accelerated cis-to-trans back isomerization under the light irradiation^18^. The thermal cis-to-trans isomerization can also be clearly seen, where the relaxation rate depends on the condensation state surrounding the chromophores. The rate constants (*k*) of the first order cis-to-trans thermal isomerization were obtained from the spectra by data-fitting (Supplementary Fig. 2). The *k* value of IA-Chol in the DMF solution is 1.6 × 10^–2^ s^−1^. IA-Chol in the solid state shows the two-stage relaxation, which have the *k* values of 4.4 × 10^–4^ s^−1^ and 6.5 × 10^–5^ s^−1^ for the thin film, and 4.3 × 10^–4^ and 7.9 × 10^–5^ s^−1^ for the submicron-pillar arrays (Supplementary Table 1). The two-stage relaxation, where the faster process is related to the strained cis isomers followed by a slower normal decay, is consistent with the previous report^41,42^. The cis-to-trans isomerization rates are similar for the solid thin film and submicron-pillar array, though, both of which are much slower compared with that of IA-Chol in the solution. The thermal relaxation rates are much slower compared with the excited-state dynamics upon light irradiation, which is in picosecond scale for *push*–*pull* type azo chromophores measured by femtosecond fluorescence and absorption techniques^43^. This observation indicates that the trans–cis isomerization is reversible, but the photo-triggered dynamic isomerization process is much faster than that of the dark cis-to-trans relaxation. The morphology and structure parameters of the IA-Chol pillar arrays were examined by the scanning electron microscopic (SEM) and atomic force microscopic (AFM) observations as well as light diffraction measurement (Supplementary Fig. 3 and 4). The submicron-pillars with the diameter (*D*) of 493 ± 9 nm and height (*H*) of 366 ± 9 nm aligned in the hexagonal lattice were adopted in the following study.

**Figure 2:**
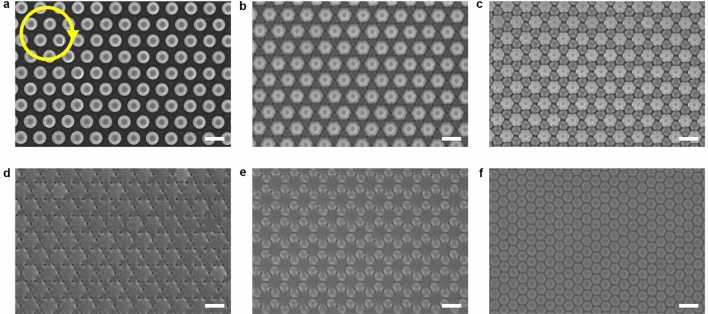
Typical top view SEM images of the IA-Chol submicron pillar arrays after being exposed to the right-hand circularly polarized light for different time periods. (**a**) 4 min. (**b**) 6 min. (**c**) 8 min. (**d**) 10 min. (**e**) 14 min. (**f**) 30 min. The yellow circular arrow represents the electric field vector pattern in the X–Y plane. The light intensity was 300 mW cm^−2^. The scale bars of all the SEM images correspond to 1 μm.

**Figure 3:**
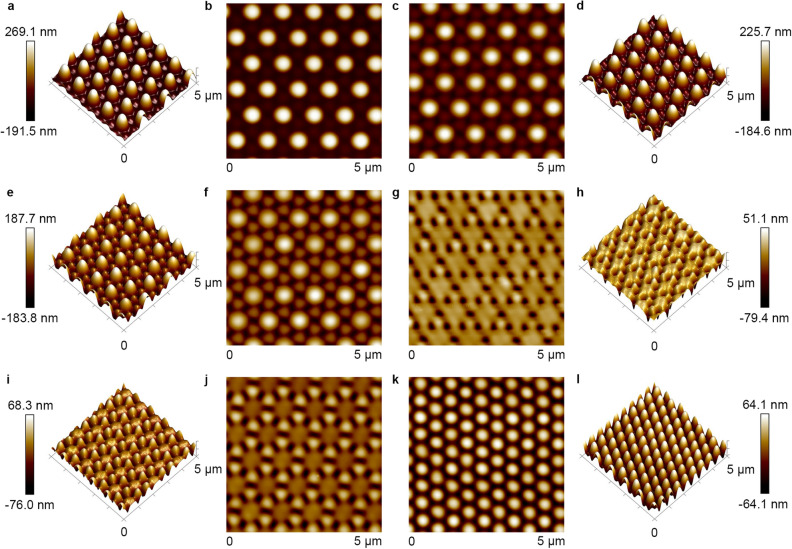
Typical AFM images of the IA-Chol submicron pillar arrays after irradiation with the right-hand circularly polarized light for different time periods. (**a, b**) 4 min. (**c, d)** 6 min. (**e, f**) 8 min. (**g, h**) 10 min. (**i, j**) 14 min. (**k, l**) 30 min. The light intensity was 300 mW cm^−2^.

To induce the topographical transition, the pillar arrays were exposed to a right-hand circularly polarized laser beam (532 nm, 300 mW cm^−2^) (Fig. 1b, Supplementary Fig. 5). The Cartesian coordinate system is selected to have the x- and y-axes in a plane parallel to the substrate and the z-axis pointing in the light propagation direction. Figure 1c shows representative images of the topographical transition when being irradiated with the right-hand circularly polarized light for different periods of time. The large-scale SEM and AFM images of the topographical transition are given in Figs. 2 and 3. In the first stage of the transition (with the light irradiation for 0–8 min), the height of the original pillars is reduced and new pillars start to emerge gradually. The height and diameter of the newly formed pillars have smaller values compared with those of the partially erased original ones in this stage. In the first half of the second stage (with the light irradiation for 9–14 min), the newly formed pillars become higher compared with the partially erased original pillars and merge with them in some parts, where the edges of the pillars become less sharp. When the light irradiation continues to 30 min, the newly-formed pillars and original ones become similar for their heights and diameters. When the light irradiation continues for over 30 min (the third stage), the final steady state manifests the well-defined hexagonal lattice of the pillars with the triple area density, which show the height and diameter of 90 ± 5 nm and 382 ± 10 nm (AFM). As shown in Supplementary Fig. 6, this effect is only related to the circular polarization of the light, which is the same for the right-hand or left-hand circularly polarized light.

### Structural evolution details in each stage

**Figure 4:**
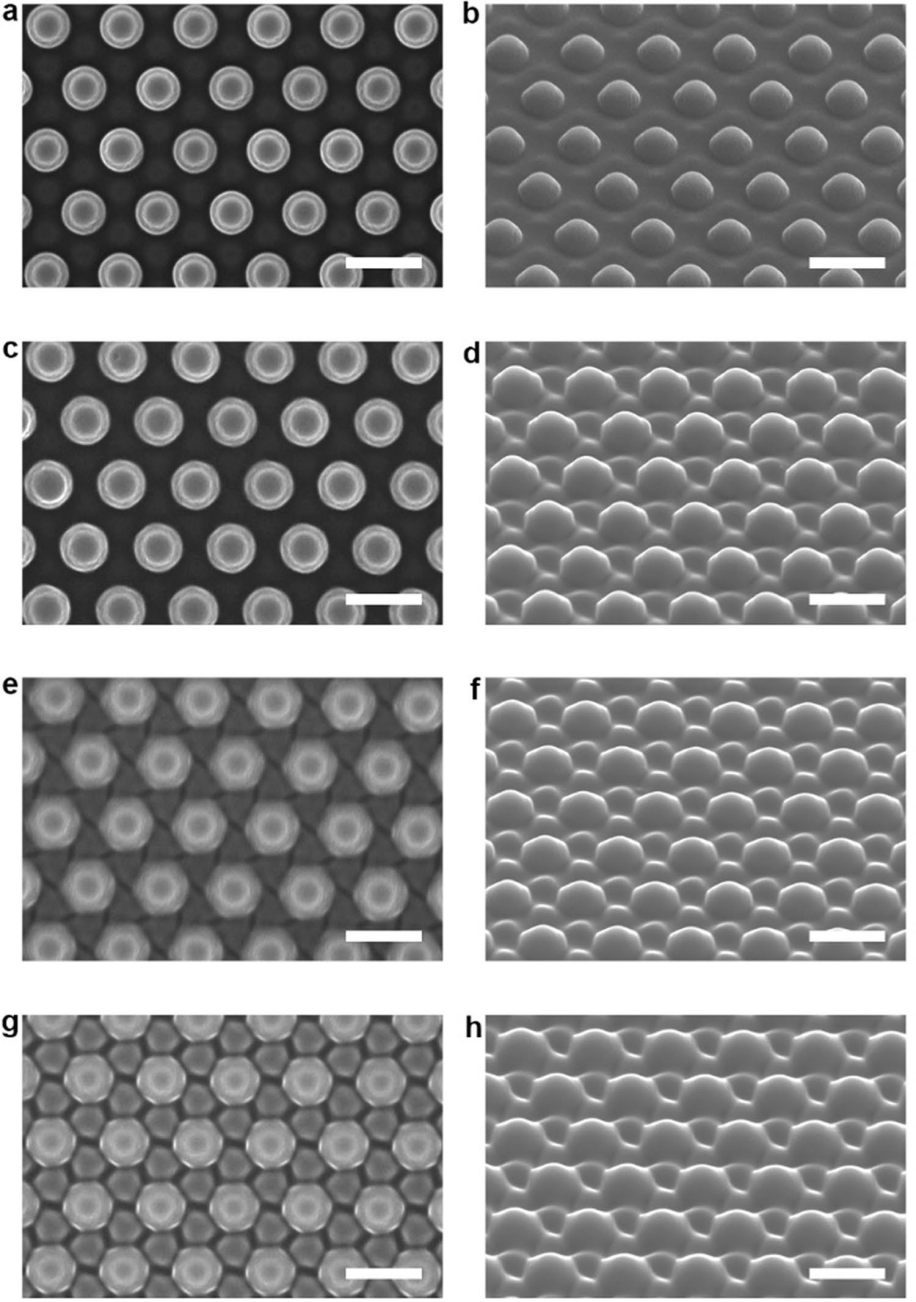
Typical SEM images of the IA-Chol submicron pillar arrays after irradiation with the right-hand circularly polarized light for different time periods. (**a**) Top-view SEM image of the array after being irradiated for 90 s. (**b**) Side-view SEM image corresponding to panel (**a**). (**c**) Top-view SEM image of the array after being irradiated for 4 min. (**d**) Side-view SEM image corresponding to panel (**c**). (**e**) Top-view SEM image of the array after being irradiated for 6 min. (**f**) Side-view SEM image corresponding to panel (**e**). (**g**) Top-view SEM image of the array after being irradiated for 8 min. (**h**) Side-view SEM image corresponding to panel (**g**). The light intensity was 300 mW cm^−2^. The scale bars of all the SEM images correspond to 1 μm.

The structural evolution in the aforementioned three stages was subjected to further SEM observation to reveal the details. In the first stage, while the exposure time was 90 s, the shallow undulating structures appear on the surface around the original pillars (Fig. 4a, b and Supplementary Fig. 7), which is caused by the effect of optical near-field^44,45^. The newly-formed pillars can be seen after being irradiated for 4 min (Fig. 4c, d). When the exposure time increases to 6 min, more complicated morphology can be seen, i.e., the newly-formed pillars with triangle cross-section and original pillars with hexagonal cross-section (Fig. 4e, f). Further increase of the exposure time to 8 min, the cross-section of newly-formed pillars also turn into hexagon as shown by the SEM images (Fig. 4g, h). As disclosed by the SEM observations, the new pillars are gradually developed with the increase of the exposure time, while the height of the original pillars decreases and the cross-section area of each original pillar becomes larger. The variations of the original pillars can be attributed to the mass transfer in the direction of electric vibration of the polarized light^28–30^, where the circularly polarized light shows the effect to erase the surface-relief structures of azo polymers and related materials^46–48^. Triangle cross-section of the newly-formed pillars and hexagonal cross-section of the original pillars observed in the intermediate stage (Fig. 4e, f) evidence the modulated light intensity due to the original pillars in array, for which the optical gradient force due to the non-uniformity of the light intensity enhances the mass transfer in the positions^27,49^. As the height of the original pillars decreases gradually in the process, to a scale obviously smaller than the light wavelength, this effect due to the modulated light intensity and the optical gradient force is no longer visible. The reason to cause the appearance of the new pillars will be discussed below.

**Figure 5:**
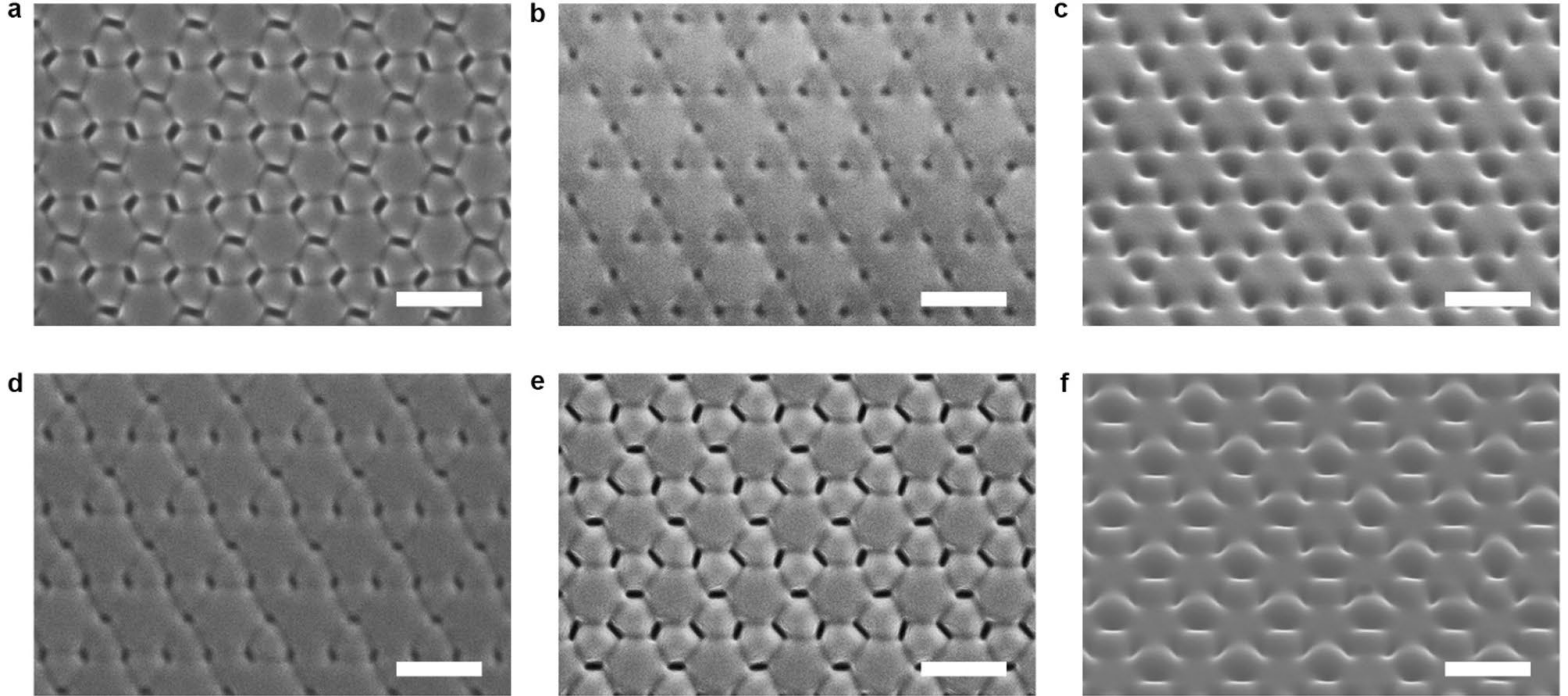
Typical SEM images of the IA-Chol submicron pillar arrays after irradiation with the right-hand circularly polarized light for different time periods. (**a**) Top-view SEM image of the array after being irradiated for 9 min. (**b**) Top-view SEM image of the array after being irradiated for 10 min. (**c**) Side-view SEM image corresponding to panel (**b**). (**d**) Top-view SEM image of the array after being irradiated for 11 min. (**e**) Top-view SEM image of the array after being irradiated for 12 min. (**f**) Side-view SEM image corresponding to panel (**e**). The light intensity was 300 mW cm^−2^. The scale bars of all the SEM images correspond to 1 μm.

**Figure 6:**
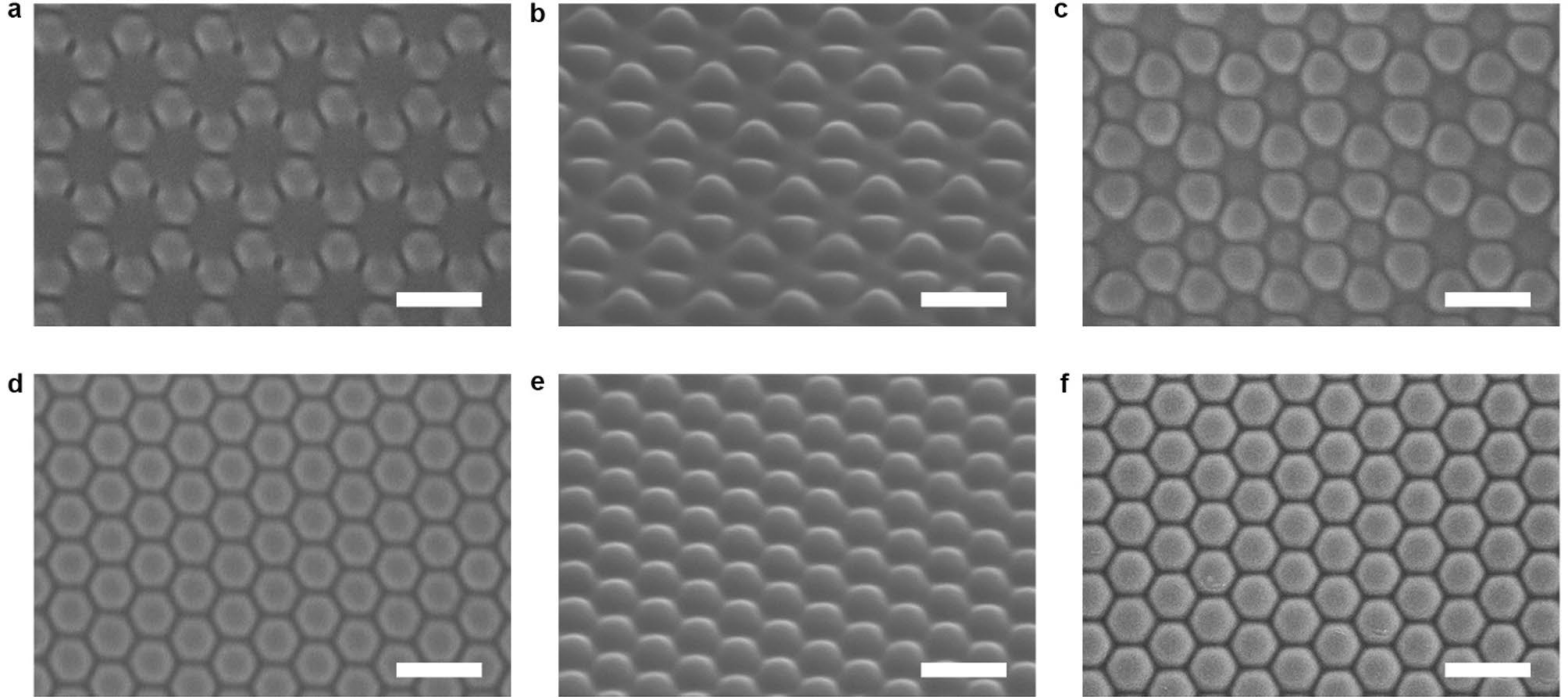
Typical SEM images of the IA-Chol submicron pillar arrays after irradiation with the right-hand circularly polarized light for different time periods. (**a**) Top-view SEM image of the array after being irradiated for 14 min. (**b**) Side-view SEM image corresponding to panel (**a**). (**c**) Top-view SEM image of the array after being irradiated for 25 min. (**d**) Top-view SEM image of the array after being irradiated for 30 min. (**e**) Side-view SEM image corresponding to panel (**d**). (**f**) Top-view SEM image of the array after being irradiated for 60 min. The light intensity was 300 mW cm^−2^. The scale bars of all the SEM images correspond to 1 μm.

**Figure 7:**
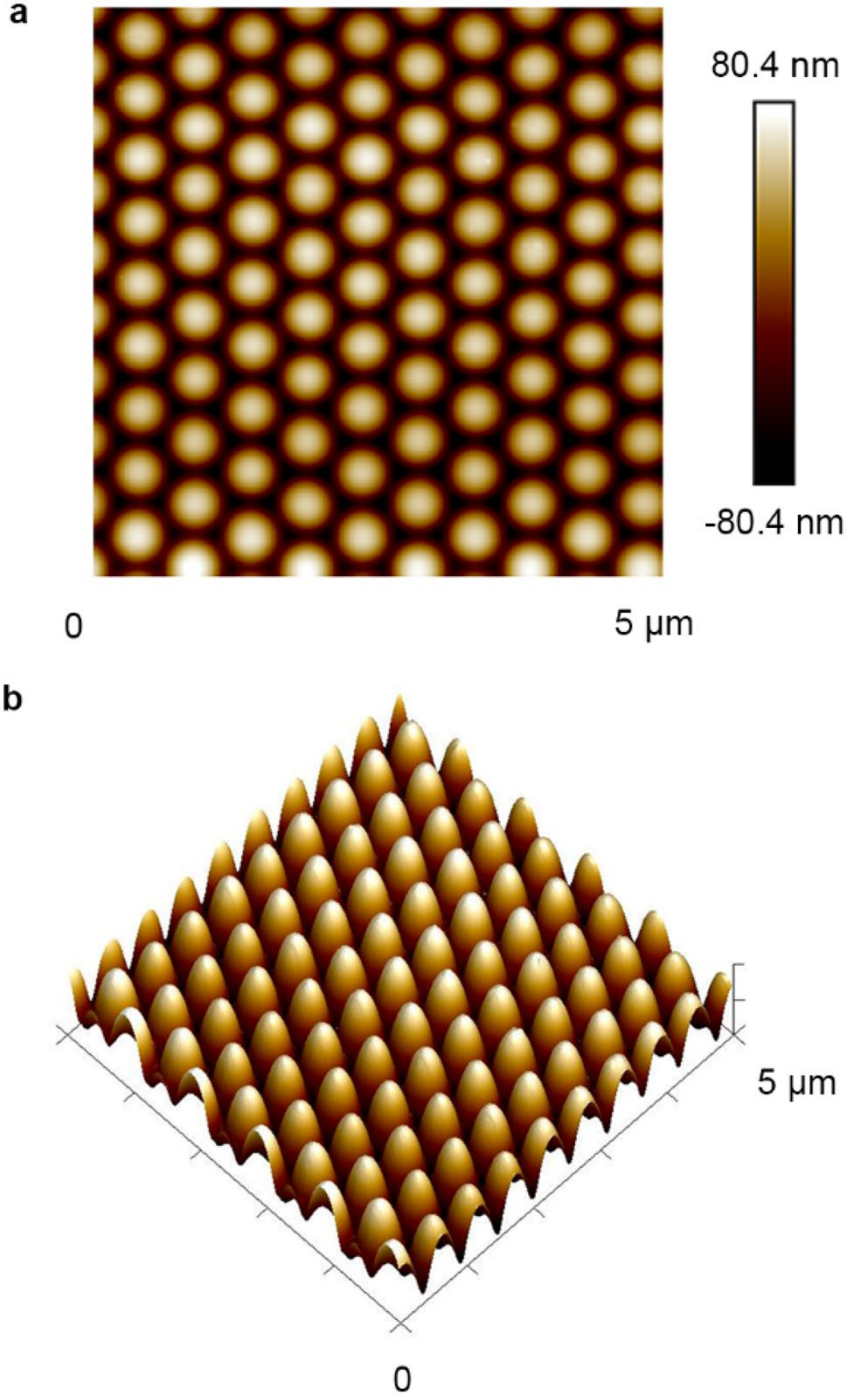
Typical AFM images of the IA-Chol submicron pillar arrays after irradiation with the right-hand circularly polarized light for 60 min. (**a**) Top-view AFM image of the array. (**b**) 3D image corresponding to panel (**a**). The light intensity was 300 mW cm^−2^.

**Figure 8:**
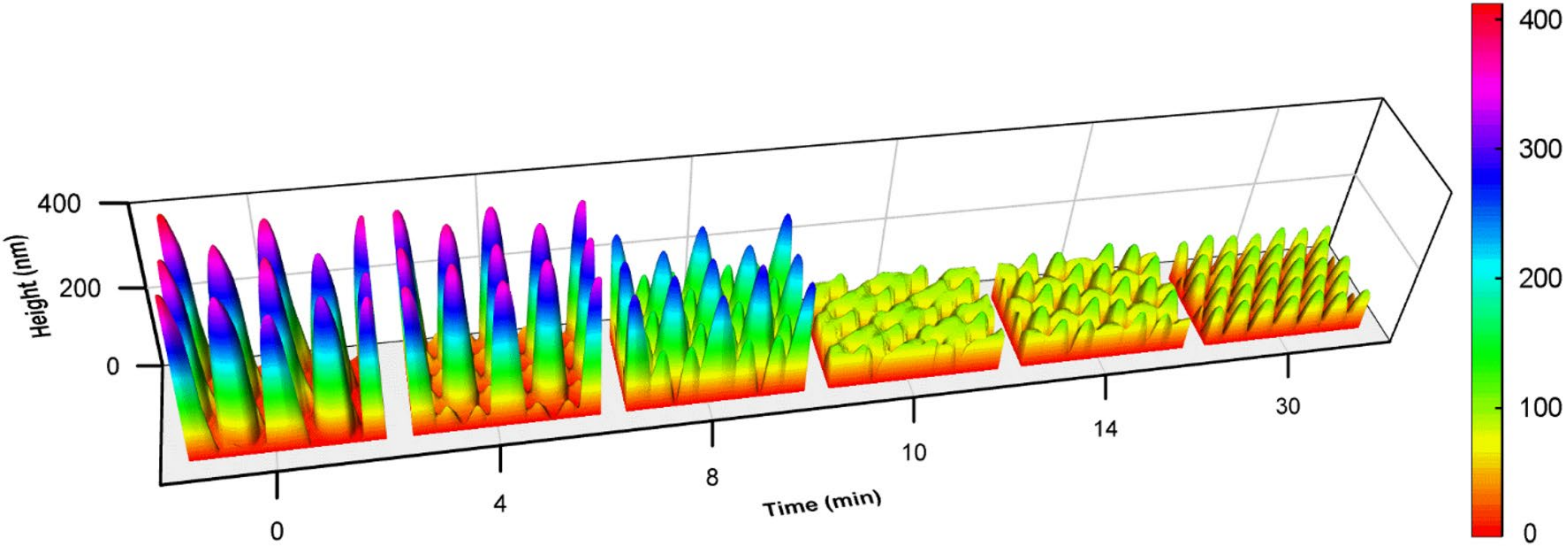
The 3D map of the IA-Chol submicron pillar array after irradiation with the right-hand circularly polarized light for different time periods. The size of the bottom area of each region is 2 μm × 3.5 μm.

**Figure 9:**
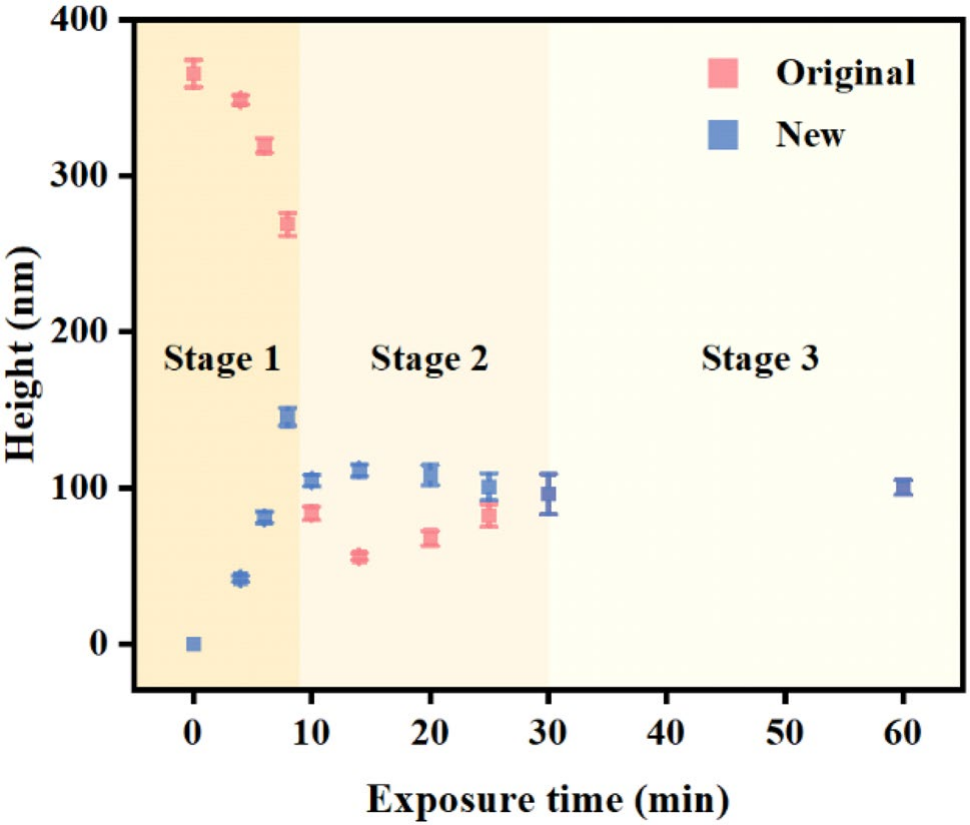
The average heights of original and newly-formed pillars versus exposure time. In the stage 1, the original pillars are higher than the newly-formed pillars. In the stage 2, the original pillars are lower than the newly-formed pillars. In the stage 3, the heights of original and newly-formed pillars are the same.

**Figure 10:**
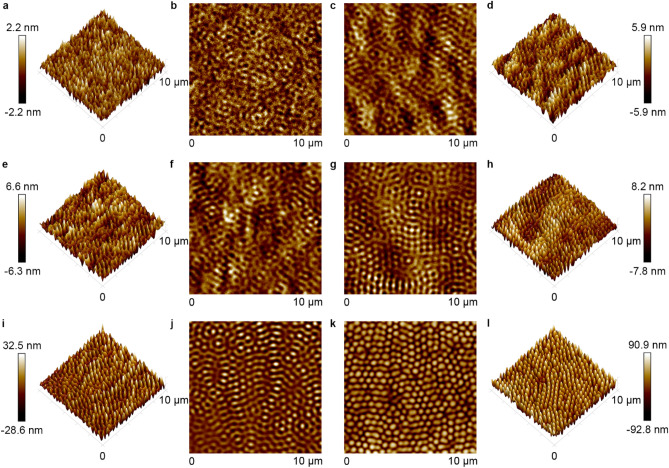
Typical AFM images of the spontaneously formed structures on IA-Chol film after irradiation with the right-hand circularly polarized light for different time periods. (**a, b**) 4 min. (**c, d**) 6 min. (**e, f**) 8 min. (**g, h**) 10 min. (**i, j**) 14 min. (**k, l**) 30 min. The light intensity was 300 mW cm^−2^.

When the height of the newly-formed pillars approaches that of the partially erased original pillars, the topographical transition enters the second stage. The change of the surface morphology can be seen from the top-view SEM images when the exposure time increases from 9 min (Fig. 5a) to 10 min (Fig. 5b). As shown in the side-view SEM image, the newly-formed and partially erased original pillars merge with each other (Fig. 5c). The surfaces of merging parts appear smooth under SEM observation, leaving some holes on the surface. SEM images show that the newly-formed pillars appear in the centers of the triangle cells of the original pillars, the holes form in the middle of each side of the triangle cells of the original pillars as a result of the mass transfer process. Since the SEM images were obtained from the secondary electron emission, the contrast is mainly dependent on the variation of the intersection angle between the incident electron beam and the normal direction of the surface to be characterized, where the small slope difference of the surface topography cannot be clearly seen in the images. As shown by AFM images (Fig. 3e–h), the more accurate picture should be that the pillars protrude on the relatively smooth surface, and the height of the newly-formed pillars surpasses that of the partially erased original pillars in the period from 8 to 10 min. With the further increase of the irradiation time to 11 min, the pillar structure becomes discernible again from SEM (Fig. 5d), which can be more clearly seen for the irradiation time of 12 min (Fig. 5e, f).

The transition from the second stage to the third stage can be clearly seen from Fig. 6a–c, which covers a process from inhomogeneous two sets of the pillar arrays to a highly uniform single pillar array. As shown in Figs. 3i, j and 6a, b (irradiation for 14 min), the newly-formed pillars are obviously higher than the partially erased original pillars, while the cross-section area of latter is larger than that of the former. With the increase of the exposure time to 25 min, all the pillars show analogous features for their heights and cross-section areas (Fig. 6c). After being irradiating for over 30 min, the uniform pillars are formed to have the height of 90 ± 5 nm and diameter at the half height of 382 ± 10 nm (AFM), which are well aligned in the hexagonal array (Fig. 6d, e). In the third stage, the formed pillar array exists in the steady state and no further change can be seen even increasing the exposure time to 60 min (Figs. 6f, 7). The correlation between the original array and the final array can be quantitatively characterized by the hexagonal 2D unit cells, obtained by projection of the pillars on the X–Y plane (Supplementary Fig. 8). The original unit cell is spanned by two primitive translation vectors **a**_1_ and **a**_2_ with the length of 995 ± 12 nm and intersection angle of 60°. The primitive translation vectors of the final pillar array (**b**_1_ and **b**_2_) have the length of 553 ± 21 nm and the intersection angle of 60°, but, both of which rotate by 30° relative to the **a**_1_ and **a**_2_. The pillars formed in the final stage are heat-erasable by increasing the temperature to 150 °C (Supplementary Fig. 9), which indicates no cross-linking reaction in the process.

### Criterions for identifying the stages

The scenario of the topographic transition with the irradiation time is illustrated by the 3D map (Fig. 8). For the light irradiation from 0 to 8 min (in the first stage), the original pillars become shorter and thicker, while the new pillars appear and grow. When the irradiation time extends from 8 to 10 min, the transition enters the second stage. Around 9 min, the heights of the newly-formed pillars and partially erased original pillars become similar and the pillars merge in some parts with each other. The details of the surface morphology can be seen in aforementioned Figs. 3e–h and 5a–c. In the middle of the second stage (14 min), the newly-formed pillars are obviously higher than the partially erased original pillars (Fig. 3i, j). When the light irradiation time extends from 14 to 30 min, all the pillars become uniform for their height and cross-section diameter. This topographical structure is stabilized and no further change can be seen when the light irradiation increases from 30 to 60 min (the third stage). The variation of the average pillar heights can be used as the criterion to classify the three stages with the irradiation time (Fig. 9). In the first stage, the height of the original pillars gradually decrease, but they are still higher than the newly formed pillars. At the beginning of the second stage, the average height of newly-formed pillars approach that of the original pillars. The height of the original pillars further decreases with the light irradiation and reaches the minimum value in the middle of the second stage and then starts to increase. In the second stage, the height of original pillars is lower than that of the newly formed pillars. In the third stage, the heights of both the newly-formed pillars and original pillars are the same, also the same are their cross-section diameters. Therefore, there is no way to distinguish whether the pillars are the original ones or new ones. As the final result, the hexagonal 2D unit cell containing three original pillars now includes nine pillars with the same heights and diameters. Besides the morphological variation (Fig. 8, Supplementary Fig. 10a and 10b), the surface areas of the relief structures also reflect the variation in the three stages. The surface area in a unit cell decreases in the first stage and then increases in the second stage, which reaches the minimum between these two stages, and keeps a stable value in the third stage (Supplementary Fig. 10c). According to the above criterions, the division between the first and second stages is around 9 min and the third stage starts from 30 min.

### Results from control experiments

To order to identify the main factors that causes the unexpected topographic transition of submicron-pillar array, the following experiments were carried out. As the decomposition temperature (*T*_d_) of IA-Chol is 276 °C, the hot embossing at 120 °C to prepare the original pillar array has no influence on the chemical structure of the material, which was confirmed by UV–vis spectra (Supplementary Fig. 1) and microscopic FT-IR spectra (Supplementary Fig. 11) of thin solid film and submicron-pillar array from the hot embossing. Thin films for preparing the submicron-pillar arrays were obtained by spin-coating the DMF solution of IA-Chol on the clean glass slides. As the films were dried at 50 °C under vacuum for 48 h before hot-embossing, no DMF remained in the solid films or submicron-pillar arrays, which was confirmed by ^1^H NMR measurement (Supplementary Fig. 12). To confirm that the topographic transition is solely related to the function of IA-Chol under light irradiation, a molecular glass (Iso-Chol) with similar isosorbide core and two peripheral cholesteryl groups, but without the azo chromophores, was synthesized and the submicron-pillar array of Iso-Chol was prepared accordingly, which are shown in Supplementary for details. As show in Supplementary Fig. 13 and Supplementary Table 2, the pillar array shows no topographic transition in contrast to that of IA-Chol pillar array under the same light irradiation condition.

## Discussion

Typical surface responses of azo polymer and azo molecular glass to light irradiation include the mass transfer in the direction of electric vibration^28–30^, and motion driven by the optical gradient force^27,49^, and related effects of optical near-field and vortex-beam illumination^41,42,50–52^. The aforementioned topographical transition induced by circularly polarized light provides deep insight into the light-matter interaction nature of this unique type of materials. As reported in previous articles, the sinusoidal surface patterns on azo polymer films can be erased optically by exposure to a polarized laser beam^46–48^. This erasing effect is attributed to the mass transfer along the direction of the electric vibration of the polarized light^28–30^. The mass transfer can be clearly seen when the IA-Chol submicron-pillar array was subjected the linearly polarized light irradiation, where the cross-section of pillars shows elongation in the direction of the electric vibration (Supplementary Fig. 14). But, as only directional mass transfer is induced by the linearly polarized light, no erasing effect similar to that upon the irradiation with the circularly polarized light can be seen. In the first stage mentioned above for the circular light irradiation, the decrease of the pillar height and increase of the diameter are caused by this effect, which is almost saturated in the second stage. The new relief structures appear simultaneously in the first stage, which becomes a dominant process in the following stages. The self-organized structure formed in the process shows some connection with another unique phenomenon reported before, i.e., the spontaneous formation of submicron-pillar patterns on the azo polymer and azo molecular glass films induced by irradiation with a single uniform laser beam^31–34^. However, there exist distinctive differences between the pattern formation on a smooth surface and from the pillar array. Figure 10 gives the surface morphology of the smooth surface of IA-Chol upon the irradiation with a circularly polarized laser beam under the same condition as that adopted for the pillar array. It can be seen that the pillar pattern starts to form for the light irradiation in few minutes (Fig. 10a–j) and enters the steady state in 30 min (Fig. 10k, l). The pillars show the height of 96 ± 13 nm, cross-section diameter at the half-height of 381 ± 21 nm and center distance between two pillars of 511 ± 29 nm in the steady state. Although these values are very similar to those of the pillars transferred from the original pillar array (Supplementary Table 3), the pillars formed on the thin film only show some local order and no long-range order can be seen. In contrast, the pillar array obtained from the topographic transition, after the light irradiation under the same condition, is arranged in hexagonal lattice with the perfect long-range order.

These observations can give valuable information to understand different mass transfer phenomena induced by the light irradiation, where the related mechanisms have not been fully elucidated at this moment. The mass transfer in the direction of the electric vibration of the polarized light is mainly manifested as the erasure effect in the first stage, which significantly reduce the height and increase the diameter of the original pillars. With the continual input of the irradiation energy, the light-penetrated layer of the surface tends to dissipate the excessive energy through heat convection through surface as much as possible and forming surface bumps to increase the surface area is favorable for heat energy dissipation. The significant decrease of the surface area of the hexagonal unit cell (*S*_c_) is attributed to the erasure of the original pillars in the first stage (Supplementary Fig. 10c), while the increase of the relief structure volume in the unit cell (*V*_c_) is caused by the emergence of the new pillars (Supplementary Fig. 15a). Then, *S*_c_ increases due to the growth of the new pillars in the second stage (Supplementary Fig. 10c), while *V*_c_ significantly decreases because of the small size of the newly-formed pillars compared with the original ones (Supplementary Fig. 15a). Therefore, *S*_c_, *V*_c_ and *S*_c_/*V*_c_ reach the extreme values between the first and second stages. In the third stage, the surface is covered by the uniform pillar array and *S*_c_/*V*_c_ reaches the maximum in favor of the heat dissipation (Supplementary Fig. 15b). The increase of *S*_c_/*V*_c_ to reach the maximum could be related to the reduced intermolecular interactions in the light-activated surface layer. When the surface adjusts its architectures to finally reach the regular and stable topography, the trans–cis photoisomerization of azo chromophores and fully activating of the surface layer are two factors playing critically important roles in the process. The first point is confirmed by the light irradiation on a polymer (CH-AN-TCV) film with similar light absorption behavior, but containing no azo chromophores, which shows no self-structured pattern formation at all (Supplementary Fig. 16). The second point is verified by the observation that the topographic transition cannot be observed for the pillar array prepared by the hot embossing on a relatively thick film of around 2.5 μm (Supplementary Fig. 17). As the transmitted light at 532 nm exponentially decays in this type of material due to absorption and only less than 20% remains at the depth of 2 μm^53^, the film between the pillars and substrate cannot be fully activated due to the rapid attenuation of the penetrated light.

Although the pillars are favorable for dissipating heat energy, increasing surface area will cause the increase of the surface tension and the new structure formation will cause the increased mechanical stress. The pillars formed on the smooth surface after the light irradiation seem to optimize the height, diameter, mutual distance in the local hexagonal lattice to reach the lowest energy. However, it does not have a factor to control the long-range order. Because of the existence of hexagonal lattice of the original pillars, the light field on the surface is modulated by the surface structures. As the self-structured pattern formation on smooth surface is enhanced by the increasing light intensity^31–34^, the newly-formed pillars appearing in the center of each triangle cell means that the light intensity is higher in these positions of the original pillar array. Moreover, in the third stage, the uniform pillars not only appear in these positions, but also appear in the positions of the original pillars. It could be attributed to the surface mobility increase at these sites in the erasing process due to deformation-induced mobility and rejuvenation^54,55^. Therefore, after the first stage light erasure, the surface has some memory of the original pattern, which guides the newly-formed pillars to be located in the original pillar positions too. The formation of highly uniform pillars is caused by the mechanism of the self-structured pattern formation to favor the energy dissipation, while the long range order of the pillars is due to the modulation of the uniform light field by original pillar array and deformation-induced mobility.

From perspective of potential applications, the surface patterning process reported here is promising due to its simplicity of one-step light irradiation. The surface patterning can be achieved on the basis of this surface topographic transition caused by the irradiation with a circularly polarized single beam. This finding has great potential to be applied for controlling morphology in the submicron scale without touching the surface.

## Methods

### Materials

Isosorbide, 4-nitrobenzoyl chloride, (*N*-ethyl-*N*-hydroxyethyl)aniline and cholesteryl chloroformate were purchased from Alfa Aesar Co. The concentrated sulfuric acid, glacial acetic acid, dichloromethane (DCM) and *N*,*N*-dimethylformamide (DMF) were purchased from commercial sources as analytical pure products. The deionized water (resistivity > 18.0 MΩ cm) was obtained from a Milli-Q water purification system. The reagents and solvents mentioned above and others were commercially available products and used as received without further purification. The PDMS molds with the periodic holes in the hexagonal lattice alignment, where the depth and diameter of the holes were 800 and 500 nm, were purchased from GD-nano Co. Ltd. The azo molecular glass (IA-Chol) was synthesized according to the method reported by us previously^56^, which is also presented in detail in the Supplementary.

### Preparation of submicron pillar arrays

The submicron pillar arrays were prepared by the soft-lithographic hot embossing^40^. The homogeneous solution was prepared by dissolving a suitable amount of IA-Chol in DMF with a typical concentration of 10 wt%, which was filtered through 0.45 μm membrane. Thin films of IA-Chol with a smooth surface were prepared by spin-coating the DMF solution on the clean glass slides (ultra-clear glass, Citotest Scientific Co., Ltd.), which were dried at 50 °C under vacuum for 48 h before use. The thickness of the films was measured by AFM and controlled to be in a range from 300 to 400 nm by adjusting the spinning rate. The PDMS molds with periodic holes on the surface were used for the hot embossing. In the process, a piece of the PDMS mold was placed and kept in seamless touch with IA-Chol film surface, heated on a hot-stage to 120 °C. The mold was then pressed downward with a gentle pressure for 1 h and the temperature was gradually lowered to 60 °C while keeping the mold in conformal touch with the IA-Chol film. After that, the PDMS mold was peeled off at 60 °C to obtain the submicron pillar arrays.

### Light irradiation setup

The optical setup used to get the circularly polarized beam was described in Supplementary Fig. 5. A linearly polarized beam from a diode-pumped frequency doubled solid state laser (532 nm, MGL-F-532, Changchun New Industries Optoelectronics Tech. Co., LTD) was used as the light source. A polarizer was placed in front of the laser to achieve the high linear polarization. The linearly polarized beam was expanded and collimated by a Galilean type beam expander. The diameter of emergent beam was 5 times as that of the incident beam. An aperture with a 2–3 mm diameter hole (significantly smaller compared with the spot size of the expanded beam) was used to get a homogeneous beam. A quarter-wave (λ/4) plate, where its fast axe was orientated by 45° to the vibration direction of the linearly polarized incoming beam, was applied to achieve the circular polarization. The circularly polarized laser beam was incident perpendicularly onto the IA-Chol submicron pillar array with the intensity of 300 mW cm^−2^ for a required time periods under ambient condition.

### Characterization

The ^1^H NMR spectra were obtained on a JEOL JNM-ECA600 NMR spectrometer with tetramethylsilane (TMS) as the internal standard in a CDCl_3_, DMSO-*d*_*6*_ or CD_2_Cl_2_ solution at 30 °C. The FT-IR spectra were collected on a Nicolet 560-IR spectrometer, where the powder samples were ground, mixed with KBr and then pressed into thin IR-transparent disks and submicron pillar array samples were measured by an IR microscope accessory (Supplementary Table 4). The morphology was characterized by a SEM instrument from Zeiss Corporation (Zeiss Merlin). A high vacuum (4 × 10^−6^ mbar approximately) condition was adopted, while the voltage and current were l5 kV and 100 pA, respectively. The surface profiles of the samples were probed using an atomic force microscope (AFM) from the Bruker Corporation (Dimension ICON-PT) in tapping mode. The diffraction pattern of the pillar array was obtained by a He–Ne laser beam incident perpendicularly on the sample.

## Supplementary Information

Supplementary Information

## Data Availability

The data supporting the findings of this study are available from the corresponding authors on reasonable request.
