# Supplementary material for: Topographical transition of submicron pillar array of azo molecular glass induced by circularly polarized light
Source: Sci Rep. 2021 Apr 1;11:7327. doi: 10.1038/s41598-021-86794-y (PMC8016868; doi:10.1038/s41598-021-86794-y)
Supplement: Supplementary file 1 — Supplementary Information [file 41598_2021_86794_MOESM1_ESM.docx]

**Supplementary Information**

Topographical transition of submicron pillar array of azo molecular glass induced by circularly polarized light

Zenan Wang, Chungen Hsu & Xiaogong Wang*

Department of Chemical Engineering, Laboratory of Advanced Materials (MOE),

Tsinghua University, Beijing 100084, People’s Republic of China.

(*Email: wxg-dce@mail.tsinghua.edu.cn)

**Table of Contents**

**Supplementary Note 1**

Synthetic scheme of the azo molecular glass (IA-Chol), p4.

Detail of each step reaction and characterization results, p5.

^1^H NMR spectrum of IA-Chol in CDCl_3_-*d_1_* with the resonance signal assignment, p8.

**Supplementary Note 2**

The synthesis of the molecular glass (Iso-Chol), p8.

Synthetic scheme of Iso-Chol, p9.

^1^H NMR spectrum of Iso-Chol in CDCl_3_-*d_1_* with the resonance signal assignment, p10.

**Characterization method**, p10

**Supplementary Figures**

Supplementary Figure 1. Variation of the UV-vis spectra of IA-Chol solution, solid thin film and submicron-pillar array induced by the light irradiation (LED, 515 nm, 200 mW cm^-2^) and relaxation at room temperature in the dark, p11.

Supplementary Figure 2. Plots of absorbance at 515 nm versus time (t) from the spectra given in Supplementary Figure 1 and fitting curves for calculating the first order *cis*-*trans* thermal isomerization, p12.

Supplementary Figure 3. Typical AFM and SEM images of the PDMS mold and submicron-pillar arrays of IA-Chol before laser irradiation, p13.

Supplementary Figure 4. The diffraction pattern and the typical image of the IA-Chol submicron pillar arrays before laser irradiation, p14.

Supplementary Figure 5. Schematic of the optical setup used to create the circularly polarized laser beam. P14.

Supplementary Figure 6. Typical top-view SEM images of the IA-Chol submicron pillar arrays after irradiation with the two orthogonally circular polarizations for 6 min, p15.

Supplementary Figure 7. Typical cross-section SEM images of the IA-Chol submicron pillar arrays at two different magnifications after irradiation with the right-handed circularly polarized light for 90 s, p15.

Supplementary Figure 8. The original and final surface topographic patterns, p16.

Supplementary Figure 9. Heat erasure of the pillars formed from the topographic transition, p17.

Supplementary Figure 10. Further quantifying information of the IA-Chol submicron pillar arrays after irradiation with the circularly polarized light for different time periods, p18.

Supplementary Figure 11. The FT-IR spectra of IA-Chol film and submicron pillar array, p19.

Supplementary Figure 12. ^1^H NMR spectrum of the sample from the IA-Chol submicron pillar array in comparison with that of DMF, p19.

Supplementary Figure 13. Typical AFM images of the submicron-pillar arrays of Iso-Chol before and after laser irradiation, p20.

Supplementary Figure 14. The SEM and AFM images of the IA-Chol pillar arrays after irradiation with the linearly polarized light, p20.

Supplementary Figure 15. Calculated surface-relief structure volume in the hexagonal cell and the specific surface area, p21.

Supplementary Figure 16. The UV-vis spectra of CH-AN-TCV in DMF and the typical AFM images of the CH-AN-TCV films before and after laser irradiation, p21.

Supplementary Figure 17. Typical AFM images of the IA-Chol submicron-pillar arrays prepared by hot embossing on the relatively thick film (thickness around 2.5 μm) before and after irradiation with the right-hand circularly polarized light for different time periods, p22.

**Supplementary Table**

Supplementary Table 1. The rate constant k of IA-Chol *cis*-*trans* thermal isomerization, p23.

Supplementary Table 2. Heights of the Iso-Chol pillars before and after the light irradiation, p23.

Supplementary Table 3. Structure parameters of the pillar arrays before and after the light irradiation, p23.

Supplementary Table 4. Analytical conditions of FT-IR, p23.

**Supplementary References**, p24.

**Supplementary Note 1**

*Synthetic scheme of the azo molecular glass (IA-Chol)*


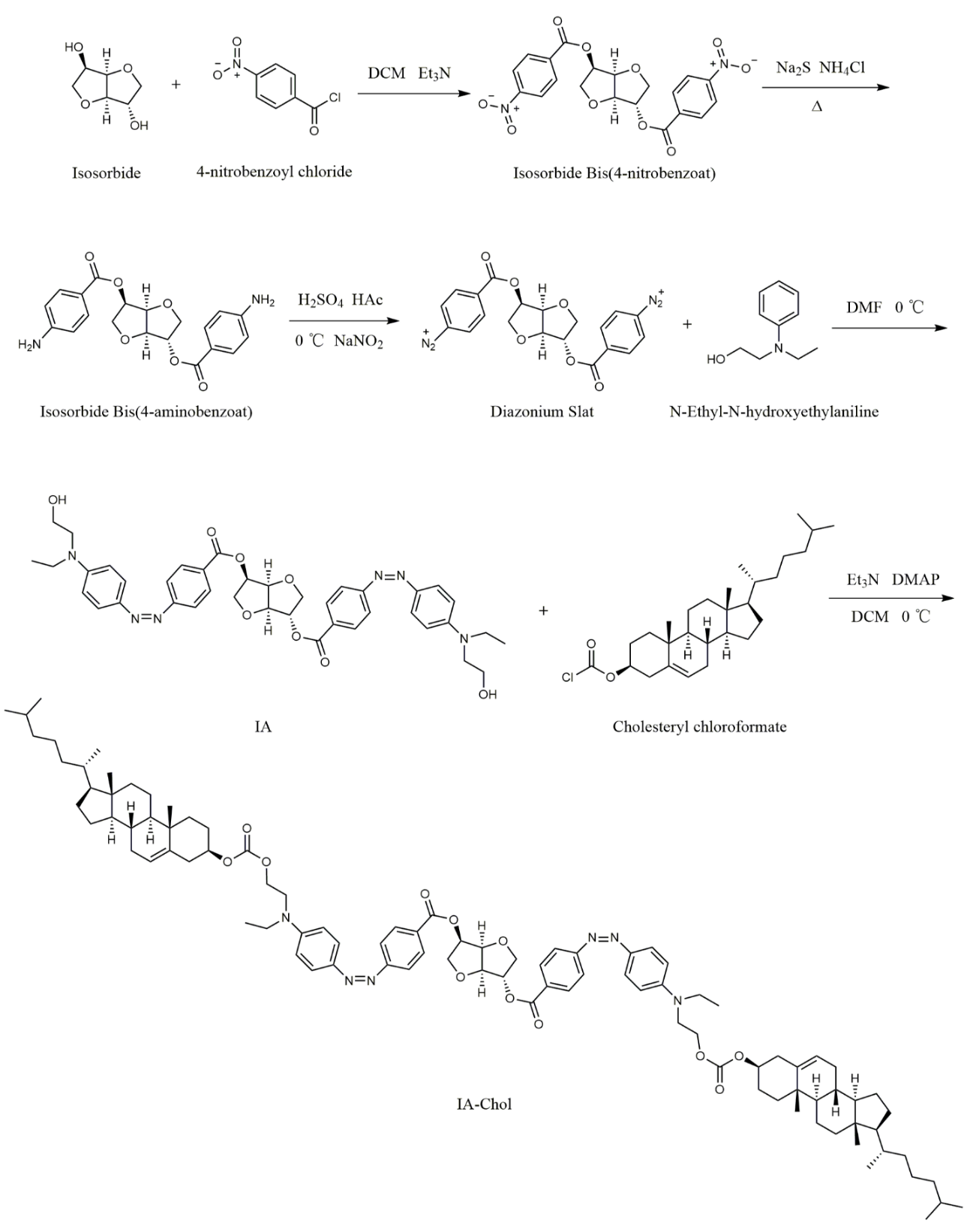


IA-Chol was synthesized according to the methods reported previously^1,2^. Details of each-step reaction are given as follows.

*Synthesis of Isosorbide Bis(4-nitrobenzoat)*


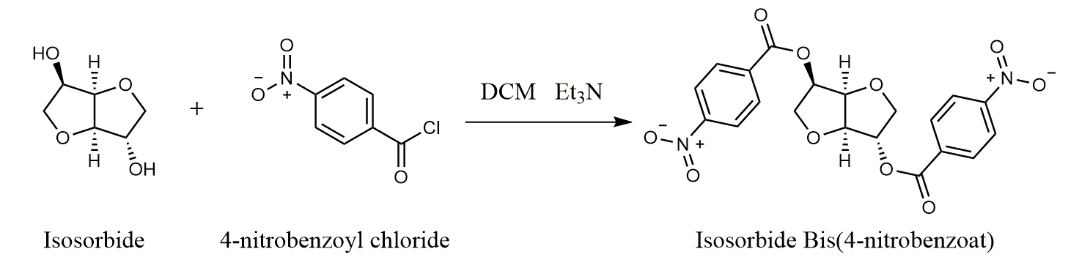


Isosorbide (7.31 g, 0.05 mol) and triethylamine (15 mL) were dissolved in CH_2_Cl_2_ (40 mL). The mixture was stirred in a round-bottom flask kept in an ice-water bath. 4-Nitrobenzoyl chloride (19 g, 0.1 mol) was dissolved in CH_2_Cl_2_ (40 mL) and dropwise added into the mixture. The reaction was carried out at the room temperature for 6 h. After the reaction, the mixture was repeatedly washed with water and the solvent was removed by rotary evaporation. The crude product was purified by column chromatography (petroleum ether/ethyl acetate = 2/1 (v/v)). Yield: 77%. MP: 131-135 °C. IR (KBr, cm^-1^): 3113 (C-H, Benz. ring, m), 1728 (C=O, s), 1606 (Benz. ring, s), 1524, 1346 (NO_2_, s), 1097 (C-O-C, s). ^1^H-NMR (600 MHz, CDCl_3_-*d_1_*, δ): 8.32-8.14 (m, 8H), 5.60-5.45 (m, 2H), 5.15-5.08 (m, 1H), 4.73-4.71 (d, 1H), 4.20-4.10 (m, 4H).

*Synthesis of Isosorbide Bis(4-aminobenzoat)*


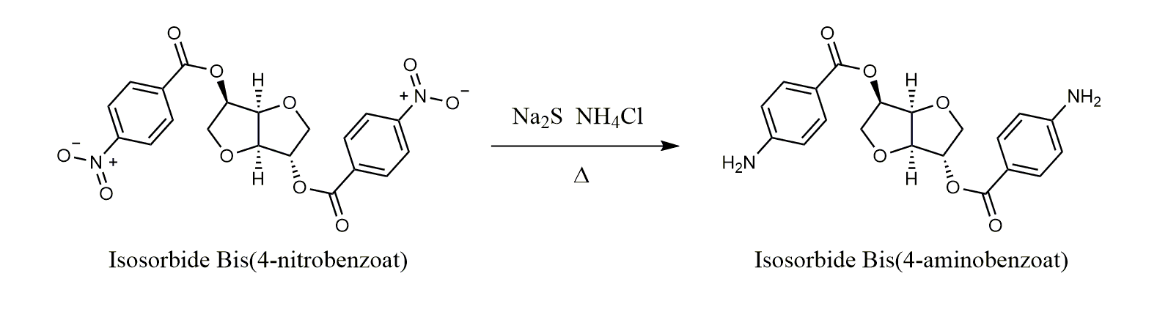


Isosorbide *bis*(4-aminobenzoat) was synthesized according to the literature^1^, where isosorbide *bis*(4-nitrobenzoat) was reduced with Na_2_S to obtain isosorbide *bis*(4-aminobenzoat). In the reaction, isosorbide *bis*(4-nitrobenzoat) (4.7 g, 10.6 mmol), Na_2_S·9H_2_O (14.4 g, 60 mmol), NH_4_Cl (4.5 g, 84 mmol), H_2_O (50 mL), and alcohol (80 mL) were added into a 250 mL round-bottom flask. The mixture was refluxed with vigorous stirring for 6 h. The precipitated powder as the impurity and the side-product was filtered out when the solution was hot. After that, the filtrate was poured into plenty of water to obtain the white precipitate, which was collected by filtration. The crude product was further purified by recrystallization in alcohol. Yield: 75%. MP: 196-198 °C. IR (KBr, cm^-1^): 3442, 3354, 3240 (N-H, s), 1689 (C=O, s), 1597, 1516 (benz. ring, s), 1279 (C-N, s), 1117 (C-O-C, s). ^1^H NMR (600 MHz, DMSO-*d_6_*, δ): 7.67-7.61 (m, 4H, ArH), 6.60-6.55 (m, 4H, ArH), 6.06-6.04 (d, 4H), 5.26-5.21 (m, 2H, isosorbide), 4.91-4.87 (m, 1H, isosorbide), 4.55-4.53 (m, 1H, isosorbide), 3.97-3.86 (m, 4H, isosorbide).

*Synthesis of IA*


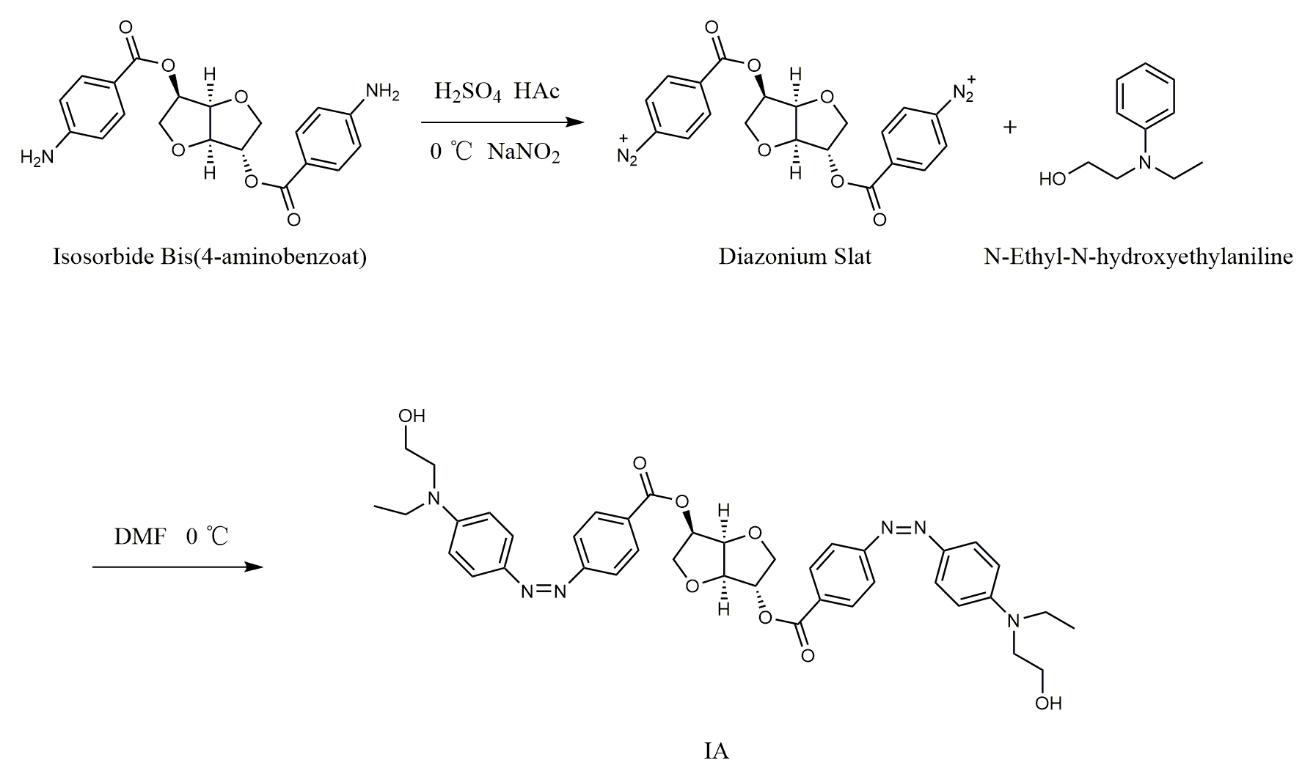


IA was synthesized according to the literature^2^. Isosorbide *bis*(4-aminobenzoat) (1.38 g, 3.6 mmol) was mixed with sulfuric acid (2 mL) and glacial acetic acid (15 mL). The diazonium salt was prepared by adding an aqueous solution of sodium nitrite (0.78 g, 11.3 mmol in 2.5 mL water) into the homogeneous mixture of isosorbide *bis*(4-aminobenzoat) with the acids. The diazonium salt, which was obtained by stirring at 0 °C until the solid was completely dissolved, was added dropwise into the DMF solution of *N*-ethyl-*N*-hydroxyethylaniline (1.49 g, 9.0 mmol). The solution was stirred at 0 °C for 12 h. After the reaction, the solution was poured into plenty of water and the precipitate was collected by filtration. The crude product was repeatedly washed with water, dried, and purified by column chromatography (petroleum ether/ethyl acetate = 1/1 (v/v)). Yield: 81%. IR (KBr, cm^-1^): 3410 (m, -OH), 2970 (CH_3_, *v_as_*), 2871, 1716 (C=O, *s_tr_*), 1596 (benz. ring, s), 1514, 1449 (benz. ring, s), 1420, 1390, 1349, 1312, 1266, 1185, 1135, 1092, 1009, 860, 821, 772, 725, 695, 541. ^1^H NMR (600 MHz, DMSO-*d_6_*, δ): 8.19-8.02 (dd, 4H, ArH), 7.97-7.71 (m, 8H, ArH), 6.96-6.76 (dd, 4H, ArH), 5.57-5.30 (m, 2H, isosorbide), 5.15-4.96 (t, 1H, isosorbide), 4.89-4.77 (s, 2H, -OH), 4.76-4.59 (d, 1H, isosorbide), 4.18-3.86 (m, 4H, isosorbide), 3.77-3.58 (t, 4H, -O-CH_2_-), 3.56-3.44 (m, 8H, -N-CH_2_-), 1.19-1.09 (t, 6H, -CH_3_).

*Synthesis of IA-Chol*


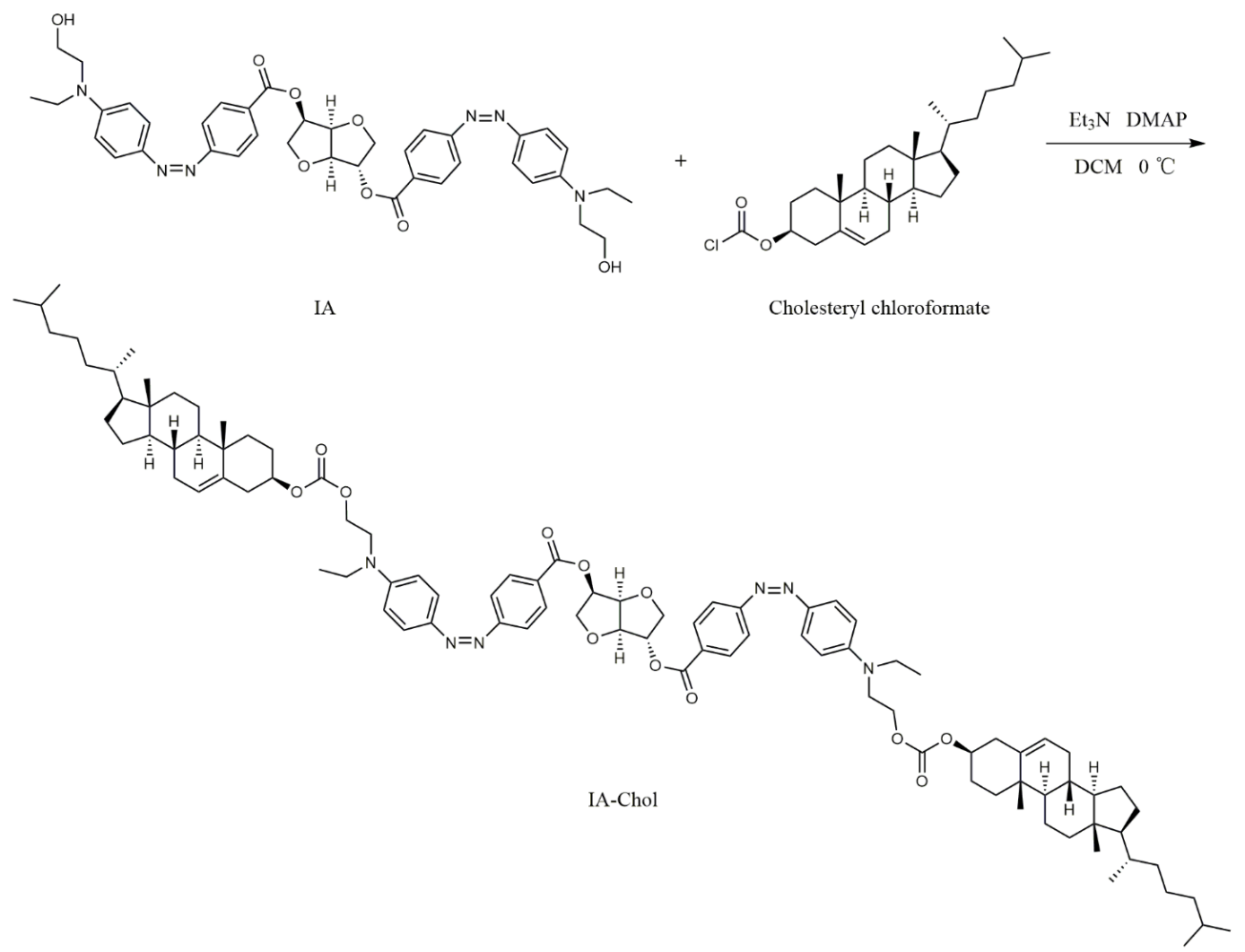


IA-Chol was prepared according to the literature^2^. IA (1.15 g, 1.6 mmol), triethylamine (0.98 g, 9.8 mmol) and 4-dimethylaminopyridine (DMAP, 0.52 g, 4.3 mmol) were dissolved in CH_2_Cl_2_ (50 mL) and then the mixture was stirred in a round-bottom flask kept in an ice-water bath. Cholesteryl chloroformate (2.18 g, 4.9 mmol) was dissolved in 30 mL CH_2_Cl_2_ and dropwise added into the flask. After the addition, the reaction was continued at the room temperature for 24 h. When the reaction was completed, the liquid with low boiling point in the reaction mixture was removed by rotary evaporation. Meanwhile, the residual mixture was washed with distilled water and extracted with CH_2_Cl_2_. The solution was dried with anhydrous MgSO_4_, and the crude product was obtained by removing the solvent through distillation and further purified by column chromatography (petroleum ether/ethyl acetate = 3/1 (v/v), the first component). Yield: 70 %. IR (KBr, cm^-1^): 2929 (CH_3_, *v_as_*), 1716 (C=O, *s_tr_*), 1596 (benz. ring, s), 1513, 1444 (benz. ring, s), 1421, 1391, 1348, 1312, 1263, 1241, 1133, 1089, 1008, 859, 820, 771, 724, 694, 539. ^1^H NMR (600 MHz, CDCl_3_-*d_1_*, δ): 8.23-8.08 (dd, 4H, ArH), 7.95-7.80 (m, 8H, ArH), 6.91-6.68 (m, 4H, ArH), 5.56-5.50 (s, 1H, isosorbide), 5.49-5.42 (s, 1H, isosorbide), 5.17-5.06 (s, 1H, isosorbide), 4.78-4.68 (s, 1H, isosorbide), 4.37-4.26 (m, 4H, isosorbide), 4.22-4.01 (t, 4H, -O–CH_2_-), 3.77-3.48 (m, 8H, -N-CH_2_-), all the other resonance signals on the ^1^H NMR spectrum represent protons of the cholesteryl group.

^1^H NMR spectrum of IA-Chol in CDCl_3_-*d_1_* with the resonance signal assignment.

**Supplementary Note 2**

*The Synthesis of the molecular glass (Iso-Chol)*

The molecular glass (Iso-Chol) as a control reference was also synthesized as follows. Isosorbide (0.29 g, 2.0 mmol), triethylamine (0.98 g, 9.8 mmol) and 4-dimethylaminopyridine (DMAP, 0.52 g, 4.3 mmol) were dissolved in CH_2_Cl_2_ (50 mL) and then the mixture was stirred in a round-bottom flask kept in an ice-water bath. Cholesteryl chloroformate (2.24 g, 5.0 mmol) was dissolved in 30 mL CH_2_Cl_2_ and dropwise added into the flask. After the addition, the reaction was continued at the room temperature for 24 h. When the reaction was completed, the liquid with low boiling point in the reaction mixture was removed by rotary evaporation. Meanwhile, the residual mixture was washed with dilute hydrochloric acid, distilled water and then extracted with CH_2_Cl_2_. The solution was dried with anhydrous MgSO_4_, and the product was obtained by removing the solvent through distillation. Yield: 89 %. IR (KBr, cm^-1^): 2950 (CH_3_, *v_as_*), 1746 (C=O, *s_tr_*), 1467, 1374, 1357, 1320, 1266, 1251, 1134, 1102, 1027, 976, 947, 885, 840, 793, 597. ^1^H NMR (600 MHz, CDCl_3_-*d_1_*, δ): 5.11-5.00 (m, 2H, isosorbide), 4.92-4.84 (t, 1H, isosorbide), 4.57-4.53 (d, 1H, isosorbide), 4.12-3.83 (m, 4H, isosorbide), all the other resonance signals on the ^1^H NMR spectrum represent protons of the cholesteryl group.

*Synthetic scheme of Iso-Chol*





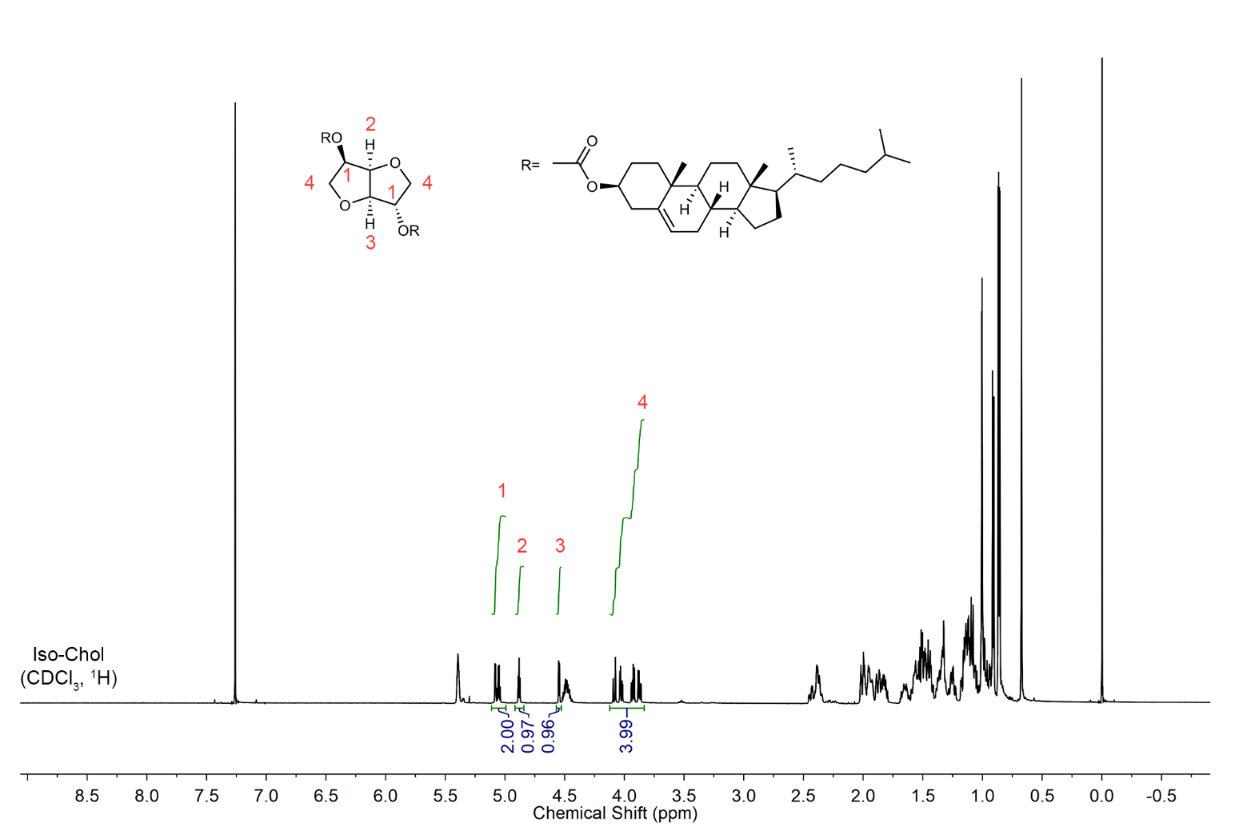


^1^H NMR spectrum of Iso-Chol in CDCl_3_-*d_1_* with the resonance signal assignment.

**Characterization method**

^1^H NMR spectra were recorded on a JEOL JNM-ECA600 NMR spectrometer (600 MHz for proton) with TMS as the internal standard in a CDCl_3_, DMSO-*d*_6_ or CD_2_Cl_2_ solution at 30 °C. FTIR spectra were obtained on a Nicolet 560-IR spectrophotometer, where the powder samples were ground, mixed with KBr and then pressed into thin IR-transparent disks and submicron pillar array samples were measured by an IR microscope accessory. The morphology was characterized by a SEM from Zeiss Corporation (Zeiss Merlin). A high vacuum (4 × 10^-6^ mbar approximately) condition was adopted, while the voltage and current were 15 kV and 100 pA, respectively. An AFM from Bruker Corporation (Dimension ICON-PT) was used to probe the surface profiles of the arrays and to measure the thickness of the films in tapping mode. The diffraction pattern was obtained via a He-Ne laser beam (633 nm) incident perpendicularly on the sample.

**Supplementary Figures**

Supplementary Figure 1. Variation of the UV-vis spectra of IA-Chol solution, solid thin film and submicron-pillar array induced by the light irradiation (LED, 515 nm, 200 mW cm^-2^) and relaxation at room temperature in the dark. (**a**) Variation of the UV-vis spectra of IA-Chol in DMF induced by the light irradiation. (**b**) Variation of the UV-vis spectra of IA-Chol in DMF in the dark. (**c**) Variation of the UV-vis spectra of IA-Chol film induced by the light irradiation. (**d**) Variation of the UV-vis spectra of IA-Chol film in the dark. (**e**) Variation of the UV-vis spectra of IA-Chol submicron-pillar array induced by the light irradiation. (**f**) Variation of the UV-vis spectra of IA-Chol submicron-pillar array in the dark.

Supplementary Figure 2. Plots of absorbance at *λ*_max_ (460 nm) versus time (t) obtained from the spectra given in Supplementary Figure 1 and fitting curves for calculating the first order *cis*-to-*trans* thermal isomerization. (**a**) IA-Chol in DMF solution (0.02 mg/mL), (**b**) ln(*A_∞_*−*A_t_*) vs *t* for IA-Chol in DMF solution (0.02 mg/mL), (**c**) IA-Chol solid thin film, (**d**) ln(*A_∞_*−*A_t_*) vs *t* for IA-Chol solid thin film, (**e**) IA-Chol submicron-pillar array, (**f**) ln(*A_∞_*−*A_t_*) vs *t* for IA-Chol submicron-pillar array.

The *cis*-to-*trans* thermal isomerization rates were calculated according to the reference by the following equation,^3,4^

$$\ln\left(A_{\infty}-A_{t}\right)=-kt+\ln\left[\left(\alpha_{t}-\alpha_{c}\right)\left[cis\right]_{0}\right]$$

where *A*_∞_ is the absorbance *A*_t_ taken at enough long time, *A*_t_ is the absorbance at *t*, *α*_t_ and *α*_c_ are two constants related to the absorbance of the *trans* and *cis* isomers at the probe wavelength, [*cis*]_0_ is *cis* isomer concentration at time 0. The rate constant (*k*) of the first order *cis*-to-*trans* thermal isomerization is obtained by plot of ln(*A*_∞_−*A*_t_) versus *t* as shown in Supplementary Figure 2b, 2d and 2f. The *k* values obtained from the data-fitting are given in Supplementary Table 1.


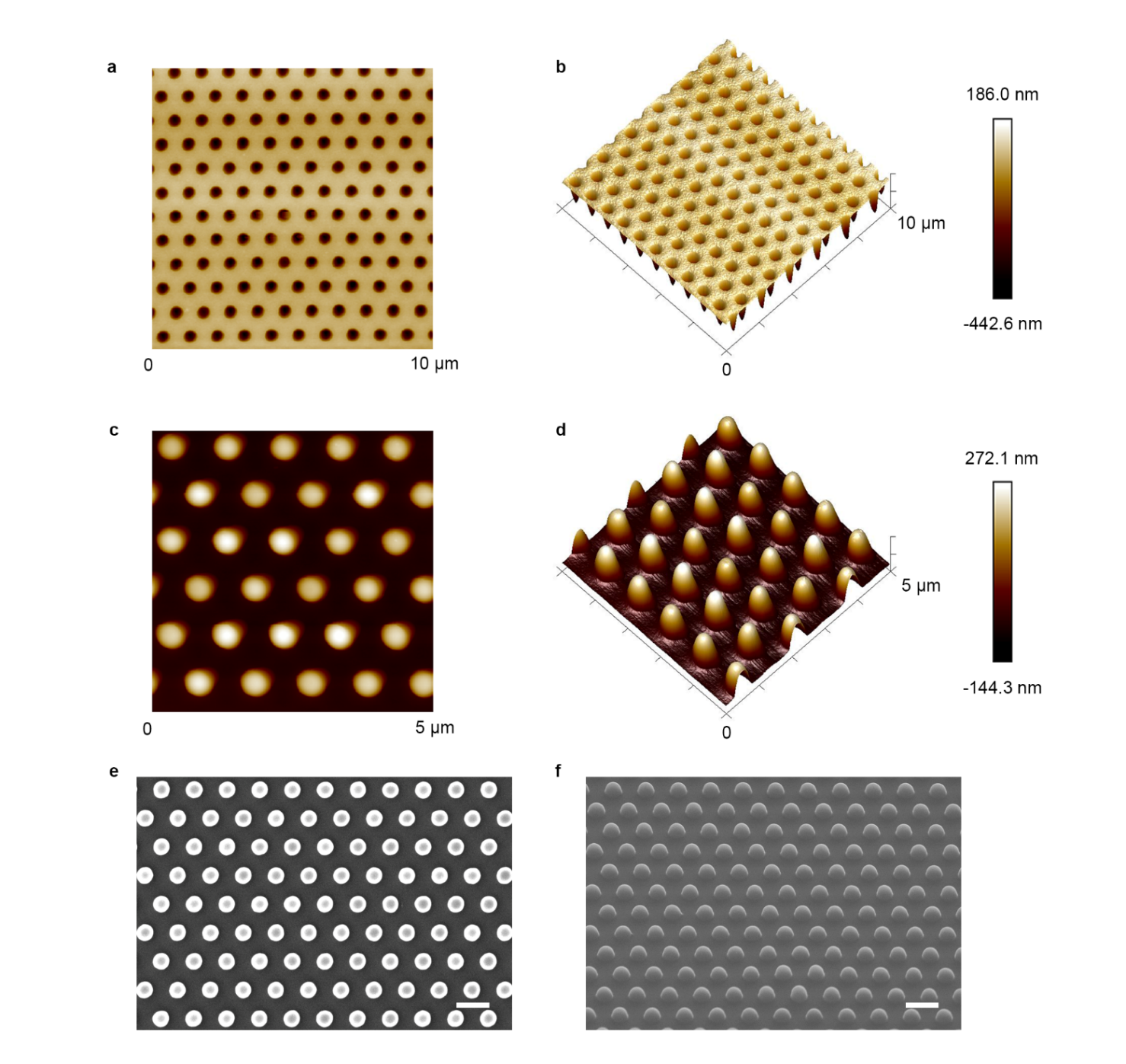


Supplementary Figure 3. Typical AFM and SEM images of the PDMS mold and submicron-pillar arrays of IA-Chol before laser irradiation. (**a**, **b**) Typical AFM images of the PDMS mold with hexagonal periodic holes. (**c**, **d**) Typical AFM images of the IA-Chol submicron-pillar array prepared by hot embossing, (**c**) top-view, (**d**) 3D-view. (**e**, **f**) Typical SEM images of the IA-Chol submicron-pillar array, (**e**) top-view, (**f**) side-view. The scale bars of the SEM images correspond to 1 μm.


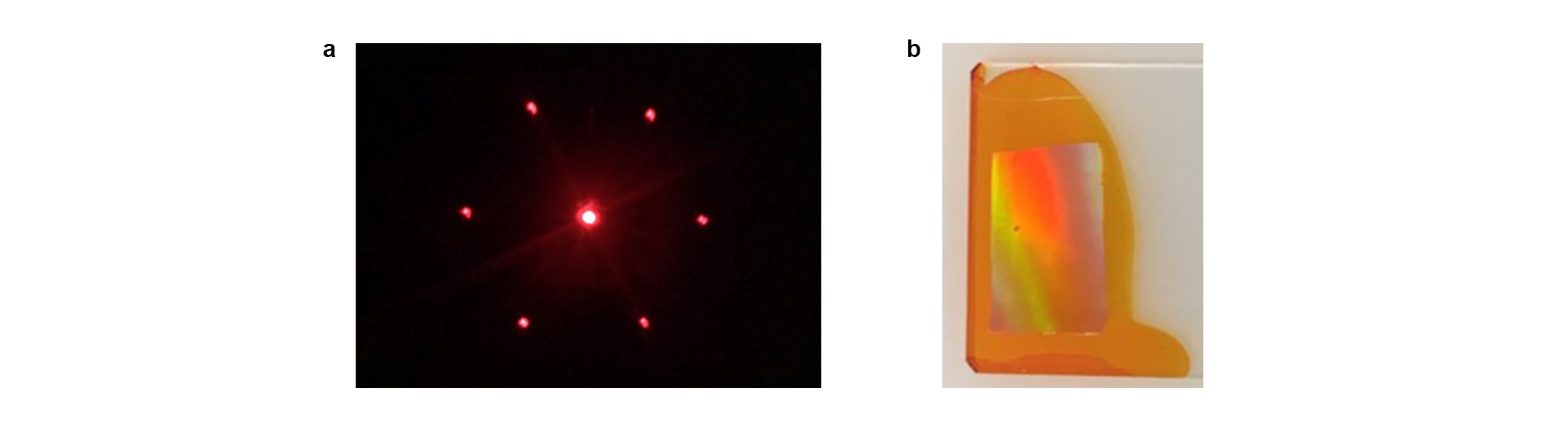


Supplementary Figure 4. The diffraction pattern and the typical image of the IA-Chol submicron-pillar arrays before laser irradiation. (**a**) The diffraction pattern of the IA-Chol submicron-pillar array before laser irradiation. (**b**) The typical image of the IA-Chol submicron-pillar array before laser irradiation. The rectangular area is covered by the pillar array and the colored stripes could be seen under the natural light.


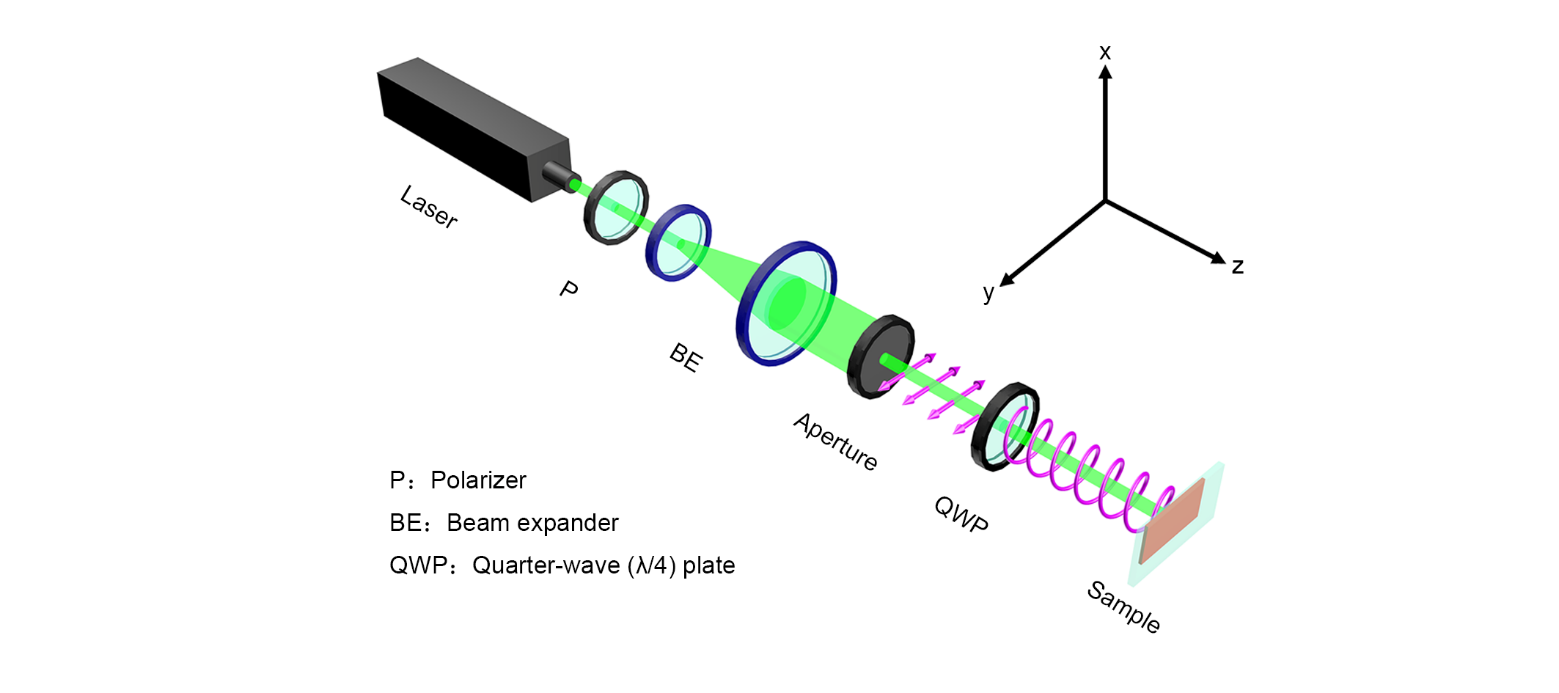


Supplementary Figure 5. Schematic of the optical setup used to create the circularly polarized laser beam. The wavelength and the intensity of the beam were 532 nm and 300 mW cm^-2^, respectively.


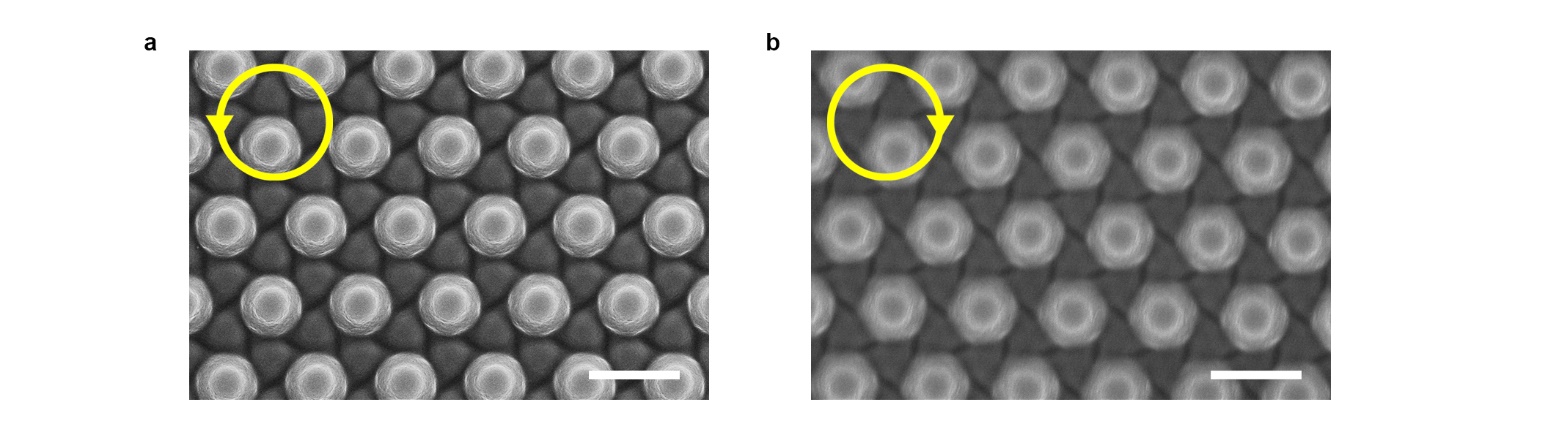


Supplementary Figure 6. Typical top-view SEM images of the IA-Chol submicron-pillar arrays after irradiation with the two orthogonally circular polarizations for 6 min. (**a**) Left-handed circularly polarized light. (**b**) Right-handed circularly polarized light. The yellow circular arrow represents the electric field vector pattern in the X-Y plane. The scale bars of the SEM images correspond to 1 μm.


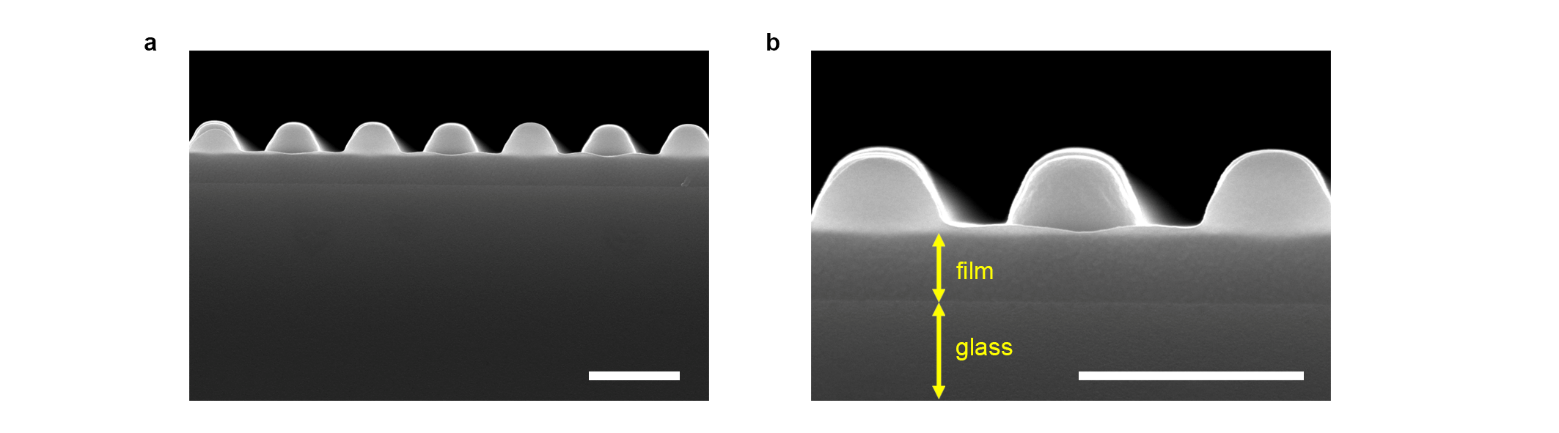


Supplementary Figure 7. Typical cross-section SEM images of the IA-Chol submicron-pillar arrays at two different magnifications after irradiation with the right-handed circularly polarized light for 90 s. (**a**) ordinary, (**b**) magnified. The scale bars in the SEM images correspond to 1 μm. The boundary between IA-Chol film under the pillars and glass substrate could be seen.


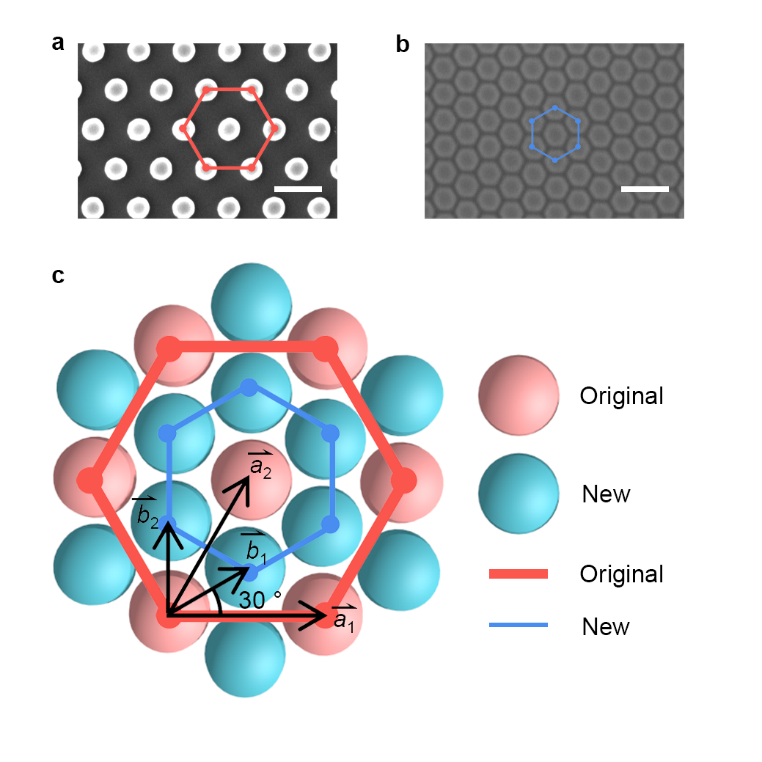


Supplementary Figure 8. The original and final surface topographic patterns. (**a**) The top-view SEM image of the IA-Chol pillar array before irradiation. (**b**) The top-view SEM image of the array after being irradiated for 60 min. (**c**) Schematic illustration of the final surface topographic patterns. The pink and light blue circles represent original and newly-formed pillars, respectively. The thick red and thin blue lines represent the original and newly-formed 2D unit cells, respectively. The light intensity was 300 mW cm^-2^. The scale bars of all the SEM images correspond to 1 μm.

Hexagonal 2D unit cells are selected to represent the original and final surface topographic patterns, which are used to illustrate the relationship between the original and the newly-formed 2D unit cells (Supplementary Figure 8c). The pink and light blue circles represent original and newly-formed pillars, respectively. The thick red and thin blue lines represent the original and newly-formed 2D unit cells, respectively (Supplementary Figure 8a, 8b). The intersection angle between the primitive translation vectors **a**_1_ and **a**_2_ of the original 2D unit cell and that of vectors **b**_1_ and **b**_2_ of the final 2D unit cell are both 60 °. Compared with the original 2D unit cell, the final 2D unit cell is rotated by 30°.


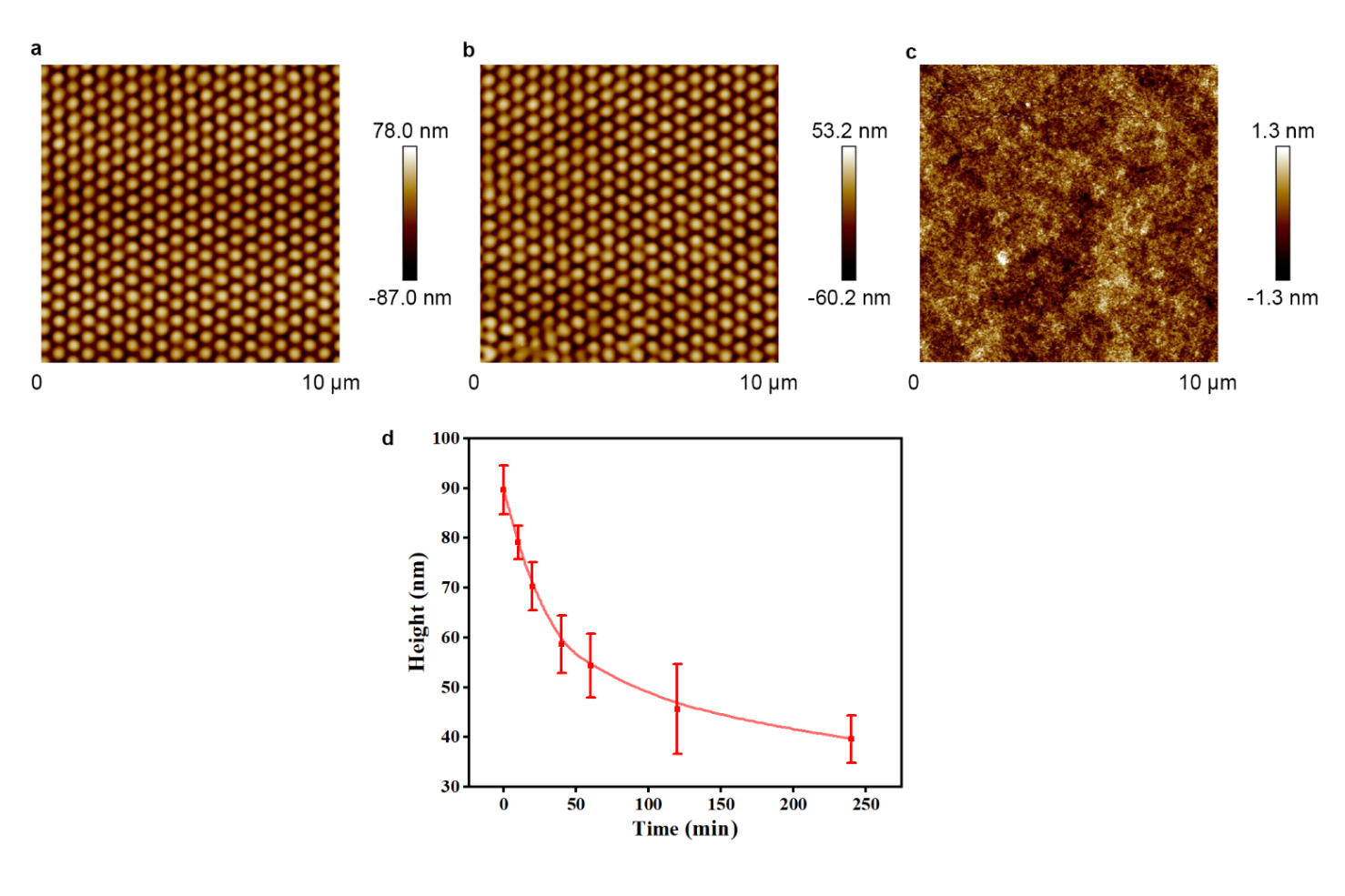


Supplementary Figure 9. Heat erasure of the pillars formed from the topographic transition. (**a**) Typical AFM image of the IA-Chol submicron pillar array obtained by irradiation with the right-handed circularly polarized light for 30 min. (**b**) Typical AFM images of the IA-Chol pillars after heating at 100 °C for 4 h. (**c**) Morphology of the surface after heating at 150 °C for another 20 min. (**d**) The height of the pillars versus heating time at 100 °C. The result shows that the pillar array formed from the topographic transition can be partially erased at 100 °C and the erasure process is significantly accelerated by increasing temperature to 150 °C.

The plane perpendicular to the substrate and parallel to the primitive translation vector **a**_1_ (Supplementary Figure 8c), containing both original and newly-formed pillars, was selected to further describe the topological transformation process (Supplementary Figure 10a). In the first stage (form orange to green), the new pillars appear and grow gradually, while the original pillars become shorter and thicker. In the second stage (blue), the height of newly-formed pillars surpasses that of original ones and the pillars partially merge with each other. In the third stage (purple), the morphology transfers from the one where the newly-formed pillars are obviously higher than the original pillars to the stable state where all the pillars are the same in their height and cross-section diameter. All of these results are consistent with the observations given in the main text. The transformation process can also be described by the parallelogram 2D cells (Supplementary Figure 10b), it could be seen that the original cell contains one pillar. The new cell contains three pillars, where one of the pillars is the original pillar (four corners of the parallelogram), and the other two are newly-formed pillars (in the central part of the parallelogram). The surface area of the hexagonal 2D unit cells (shown by the thick red lines in Supplementary Figure 8c) versus the irradiation time is given Supplementary Figure 10c. The unit cell surface area shows a trend of decrease in the first stage, increase in the second stage and being stabilized in the third stage.


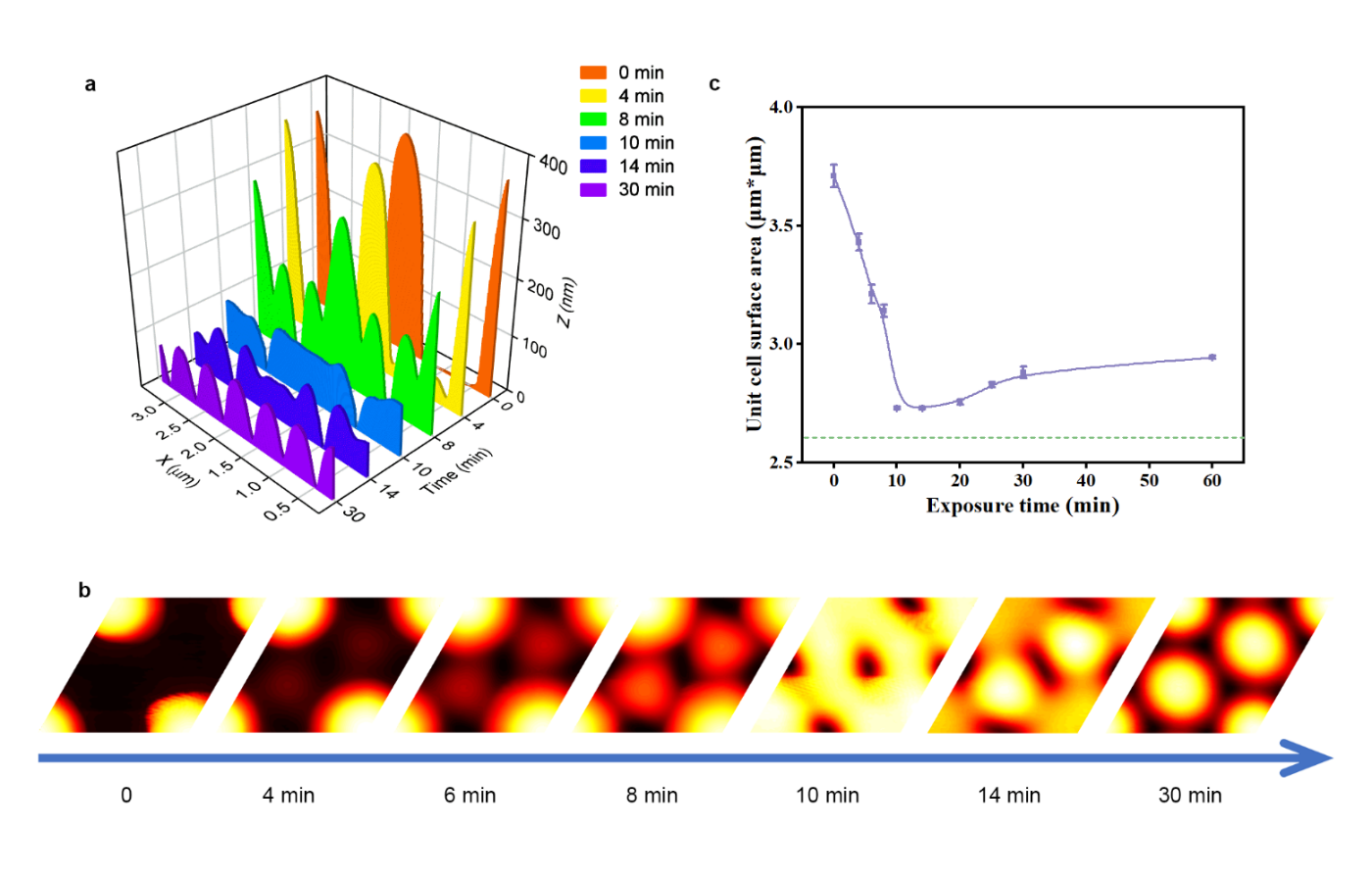


Supplementary Figure 10. Further quantifying information of the IA-Chol submicron-pillar arrays after irradiation with the circularly polarized light for different time periods. (**a**) The cross-section view of the plane parallel to vector **a**_1_ after being irradiated for different time periods. (**b**) Top-view image of the 2D parallelogram cell after being irradiated for different time periods. (**c**) The relationship between the surface area of the hexagonal unit cell and exposure time (solid line), where the surface area of a smooth plane surface is given by dashed line for comparison. The curve in the figure is used for the eye-guiding purpose. The light intensity was 300 mW cm^-2^.





Supplementary Figure 11. The FT-IR spectra of IA-Chol film and submicron pillar array. The blue line (below) represents the FT-IR spectrum of IA-Chol film. The red line (above) represents the FT-IR spectrum of IA-Chol submicron-pillar array. In this analysis process, thin solid IA-Chol film was obtained by spin-coating on the low-E glass (S. T. Japan Inc.) with its DMF solution and drying appropriately. The pillar array was prepared by hot embossing on the IA-Chol film. The FT-IR spectra were collected by a FT-IR microscope accessory (Shimadzu AIM-9000) with conditions shown in Supplementary Table 4.


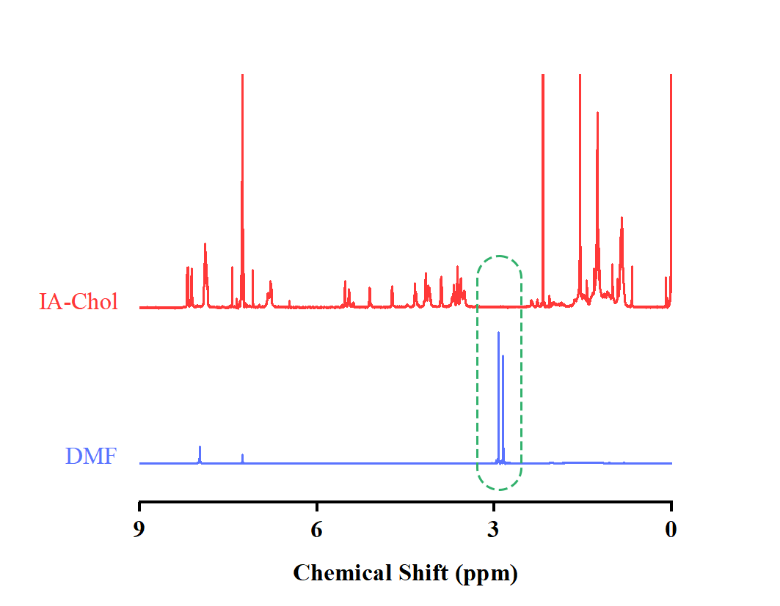


Supplementary Figure 12. ^1^H NMR spectrum of the sample from the IA-Chol submicron-pillar array in comparison with that of DMF. (**Top**) The sample from the IA-Chol submicron pillar array dissolved in CDCl_3_-*d_1_*. (**Bottom**) ^1^H NMR spectrum of DMF dissolved by CDCl_3_-*d_1_* (10 mg mL^-1^). The green dotted line marks the resonance signal of DMF and there is no DMF remained in the pillar array.


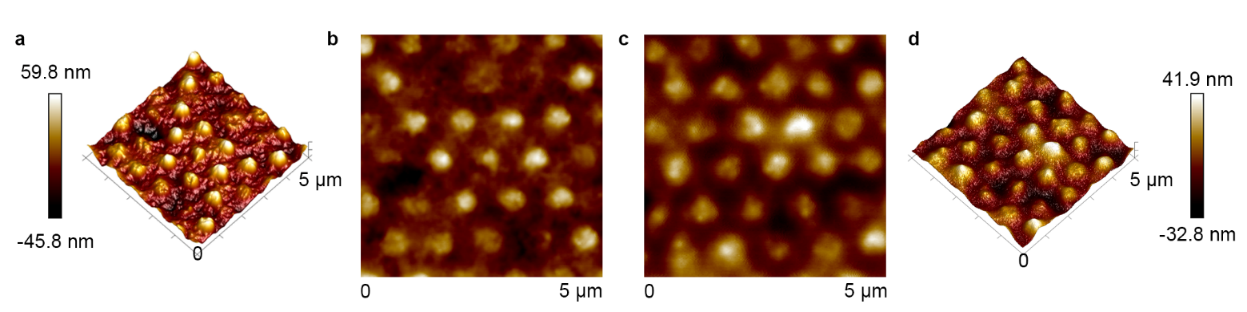


Supplementary Figure 13. Typical AFM images of the submicron-pillar arrays of Iso-Chol before and after laser irradiation. (**a**, **b**) Typical AFM images of the Iso-Chol submicron pillar array prepared by hot embossing before laser irradiation. (**c**, **d**) Typical AFM images of the Iso-Chol submicron pillar array after irradiation with the right-handed circularly polarized light for 60 min. Although the hot embossing could not produce the pillar array as regular as that of IA-Chol, which is stemmed from the good processing ability of IA-Chol but it is lacking for Iso-Chol, the result proves that the pillar array of Iso-Chol shows no variation upon the light irradiation.


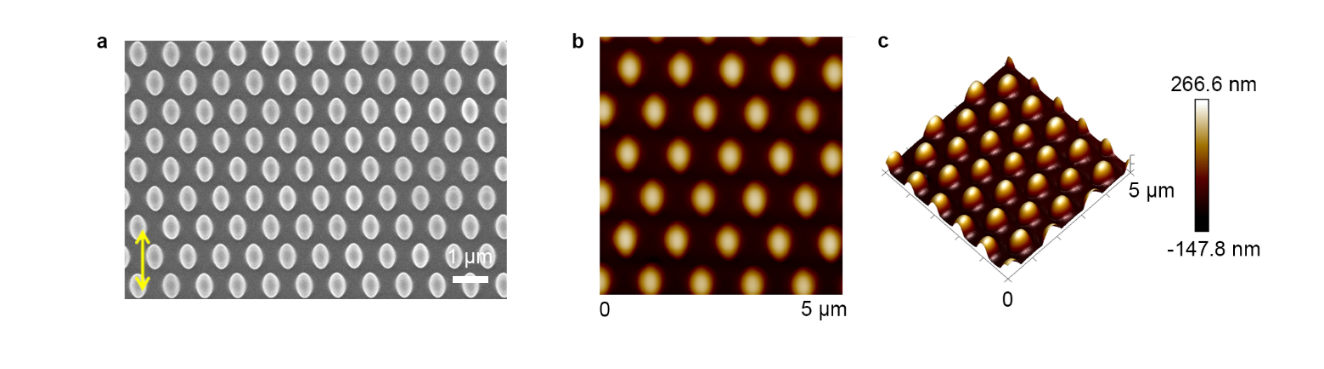


Supplementary Figure 14. The SEM and AFM images of the IA-Chol pillar arrays after irradiation with the linearly polarized light. (**a**) The top-view SEM image of the IA-Chol pillar array after irradiation with the linearly polarized light for 2 min. The yellow double-sided arrow represents the electric vibration direction of the polarized light in the X-Y plane. The scale bar corresponds to 1 μm. (**b**, **c**) Typical AFM images of the IA-Chol submicron pillar arrays after irradiation with the linearly polarized light for 2 min. The light intensity was 300 mW cm^-2^.

Supplementary Figure 15. Calculated surface-relief structure volume in the hexagonal unit cell and the specific surface area. (**a**) The surface-relief structure volume in the cell for the IA-Chol submicron pillar arrays after irradiation with the right-handed circularly polarized light for different time periods. (**b**) The ratio of surface area (*S*_c_) in the hexagonal cell to the surface-relief structure volume (*V*_c_) versus time for the irradiation with the right-handed circularly polarized light. All curves in the figures are used for the eye-guiding purpose.


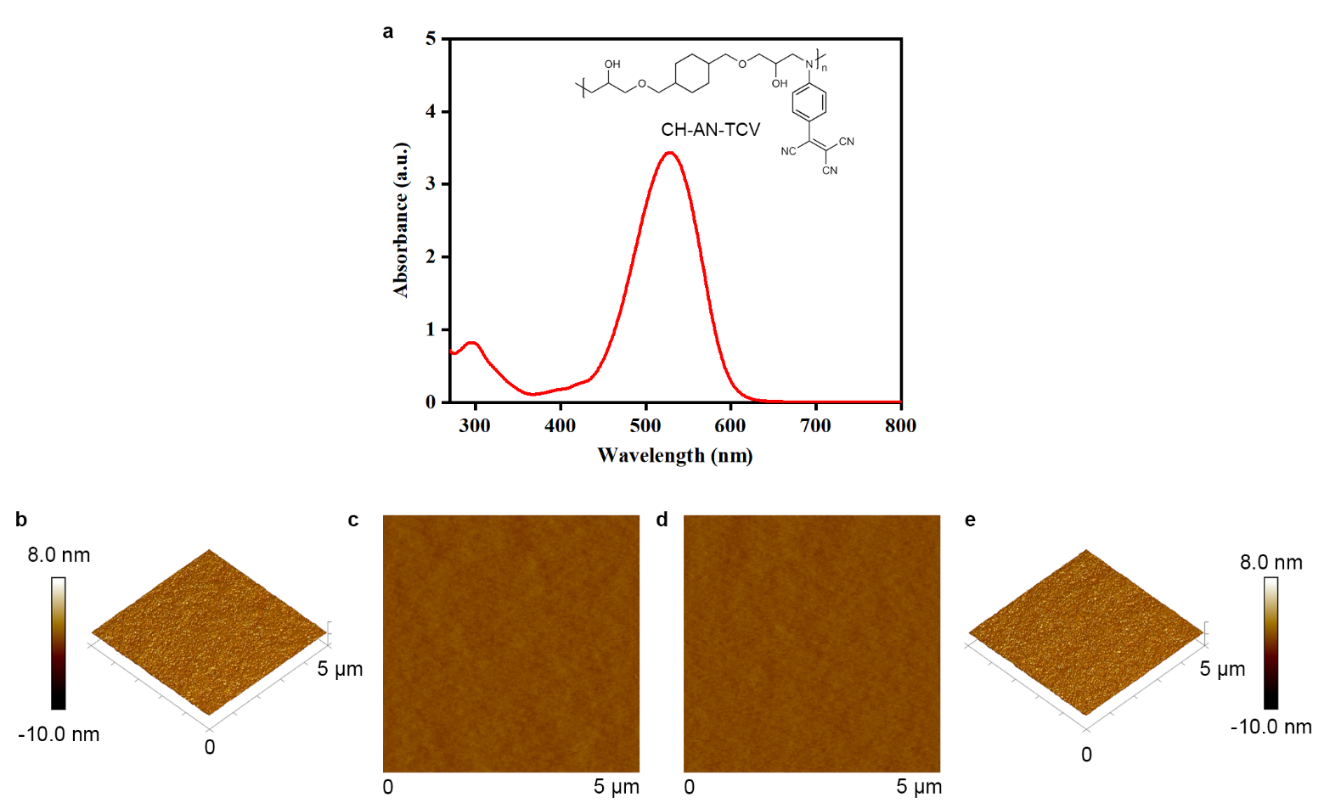


Supplementary Figure 16. The UV-vis spectra of CH-AN-TCV in DMF and the typical AFM images of the CH-AN-TCV films before and after laser irradiation. (**a**) The UV-vis spectrum of CH-AN-TCV in DMF. (**b, c**) Typical AFM images of the CH-AN-TCV film prepared by spin-coating before laser irradiation. (**d**, **e**) Typical AFM images of the same CH-AN-TCV film after irradiation with the right-handed circularly polarized light for 60 min.


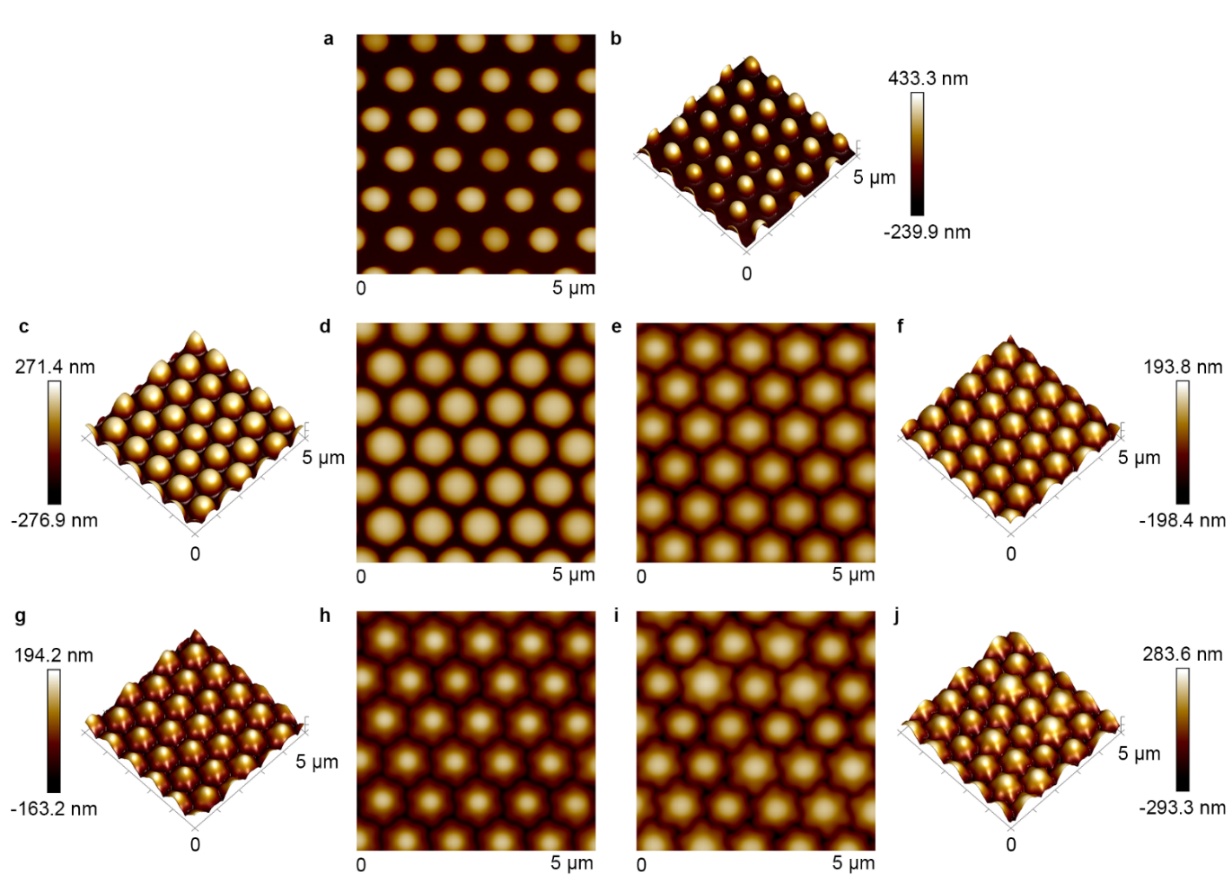


Supplementary Figure 17. Typical AFM images of the IA-Chol submicron-pillar arrays prepared by hot embossing on the relatively thick film (thickness around 2.5 μm) before and after irradiation with the right-handed circularly polarized light for different time periods. (**a**, **b**) Typical AFM images of the IA-Chol submicron-pillar arrays before the irradiation. (**c-j**) Typical AFM images of the IA-Chol submicron-pillar arrays after irradiation with the right-handed circularly polarized light for 5 min (**c**, **d**), 10 min (**e**, **f**), 20 min (**g**, **h**) and 60 min (**i**, **j**). The light intensity was 300 mW cm^-2^

**Supplementary Table 1.** The rate constant *k* of IA-Chol *cis*-to-*trans* thermal isomerization

| IA-Chol State | *k* (s^-1^) | |
| --- | --- | --- |
| DMF Solution (0.02 mg/mL) | 1.6 × 10^-2^ ± 3.7 × 10^-3^ | |
| Solid Thin Film | 4.4 × 10^-4^ ± 4.4 × 10^-5^ | 6.5 × 10^-5^ ± 9.4× 10^-6^ |
| Submicron-pillar Array | 4.3 × 10^-4^ ± 5.0 × 10^-5^ | 7.9 × 10^-5^ ± 1.2× 10^-5^ |

**Supplementary Table 2.** Heights of the Iso-Chol pillars before and after the light irradiation.

|  | Pillar before Irradiation | Pillar after Irradiation for 60 min |
| --- | --- | --- |
| Height/nm | 40 ± 15 | 36 ± 8 |

**Supplementary Table 3.** Structure parameters of the pillar arrays before and after the light irradiation.

|  | Pillar before Irradiation | Pillar after Irradiation for 30 min | Film after Irradiation for 30 min |
| --- | --- | --- | --- |
| Height/nm^a^ | 366 ± 9 | 90 ± 5 | 96 ± 13 |
| Diameter/nm^b^ | 493 ± 9 | 382 ± 10 | 381 ± 21 |
| Distance/nm^c^ | 995 ± 12 | 553 ± 21 | 511 ± 29 |

Notes: a. Heights of the pillars, b. cross-section diameters of the pillars at the half-height, c. distances between the centers of the adjacent pillar.

**Supplementary Table 4.** Analytical conditions of FT-IR

| Attenuated Total Reflection | Infrared Microscope: Shimadzu AIM-9000 |
| --- | --- |
| Wavenumber Range | 700 - 4000 cm^-1^ |
| Resolution | 4 cm^-1^ |
| Cumulated Number | 32 scans |
| Aperture Size | 10 × 10 μm |
| Analysis Software | LabSolutions IR |

**Supplementary References**

1. Guo, M. C., Xu, Z. D. & Wang, X. G. Photofabrication of Two-Dimensional Quasi-Crystal Patterns on UV-Curable Molecular Azo Glass Films. *Langmuir* **24**, 2740-2745 (2008).

2. Hsu, C. E., Xu, Z. D. & Wang, X. G. Holographic Recording and Hierarchical Surface Patterning on Periodic Submicrometer Pillar Arrays of Azo Molecular Glass via Polarized Light Irradiation. *Adv. Funct. Mater.* **28**, 15 (2018).

3. Barrett, C., Natansohn, A. & Rochon, P. Thermal Cis-Trans Isomerization Rates of Azobenzenes Bound in the Side Chain of Some Copolymers and Blends. *Macromolecules* **27**, 4781-4786 (1994).

4. Barrett, C., Natansohn, A. & Rochon, P. Cis-Trans Thermal Isomerization Rates of Bound and Doped Azobenzenes in a Series of Polymers. *Chem. Mater.* **7**, 899-903 (1995).
